# Hybrid channel structure and product quality distribution strategy for online retail platform

**DOI:** 10.1371/journal.pone.0285860

**Published:** 2023-05-18

**Authors:** Qi Zhang, Huaxin Liu, Zigong Cai

**Affiliations:** 1 Management School, Hainan University, Haikou, China; 2 School of Civil and Transportation Engineering, Henan University of Urban Construction, Pingdingshan, China; Wuhan Textile University, CHINA

## Abstract

Currently, platforms (such as Amazon.com and JD.com) are gradually transitioning from pure resellers to platforms providing hybrid channel structures. In a hybrid channel, the reselling channel and the agency channel on the platform are simultaneously used. Therefore, according to the agent who sells through the agency channel (manufacturer or third-party retailer), two kinds of hybrid channel structurers can be selected by the platform. At the same time, due to the intense competition caused by the hybrid channel structure, platforms take the initiative to implement the product quality distribution strategy in which different quality products are sold through various retailing channels. Thus, from the perspective of platforms, how to coordinate the selection of hybrid channel structures and the implementation of the product quality distribution strategy is a significant issue that has been overlooked in existing literature. This paper proposes game-theoretic models to investigate whether a platform should choose “which” hybrid channel structure to use and decide “whether” to adopt the product quality distribution strategy. Our analysis shows that the game equilibrium is affected by the commission rate, the product differentiation level, and the production cost. More specifically, first, it is interestingly found that if the product differentiation level exceeds a particular threshold, the product quality distribution strategy can negatively influence the retailer to abandon the hybrid retailing mode. In contrast, the manufacturer continues to sell through the agency channel as a part of the product distribution plan. Second, regardless of the channel configuration, we find that the platform chooses to increase the order quantity with the help of the product distribution plan. Third, contrary to popular belief, the product quality distribution strategy can only benefit the platform when the third-party retailer participates in hybrid retailing with a suitable commission rate and product differentiation level. Fourth, the platform should make decisions on the above two strategies concurrently; otherwise, agency sellers (manufacturer or third-party retailer) will oppose the product quality distribution strategy. Our key findings can assist stakeholders in making strategic decisions about hybrid retailing modes and product distribution.

## Introduction

With the growth of the internet, in recent decades, various promising e-commerce platforms have emerged in many countries. Reselling, through which platforms order products directly from manufacturers and sell them to consumers, is generally used by many e-commerce companies, such as Amazon.com and JD.com. However, with the growth of the number of e-retailers, a second kind of channel structure (i.e., agency retailing) is increasingly energetic. Traditional reselling platforms also want a piece of the pie from the growth of e-retailers. Therefore, platform companies propose a hybrid channel structure that allows third-party retailers and manufacturers to sell straight to customers on their platforms through an agency channel with a category-based commission. Meanwhile, with more intense channel rivalry, platforms are focusing more on how to maintain their competitive advantages. The product quality distribution strategy, in which third-party retailers and manufacturers should sell products of different quality than the one sold by the platform, is an option for the platforms. Thus, when the platforms establish a hybrid channel structure, two kinds of problems should be solved; these include (1) whether the third-party retailer or the manufacturer is a better choice for the agency channel and (2) whether the product quality distribution strategy should be used in the hybrid channel structure.

In real situations, the development of logistics networks and the lower operating costs of platforms encourage offline retailers to open stores and sell products on platforms. As hybrid retailing modes evolve, an increasing number of manufacturers and third-party retailers sell through the agency channel. For example, JD.com, one of the leading e-commerce platforms in China, has agreed to allow many manufacturers and third-party retailers to sell products. According to reports, the number of third-party shops surpassed 250,000 in the third quarter of 2019. For Amazon.com, in the third quarter of 2020, 54% of total revenue was derived from the agency channel. During the agency channel development process, platforms publish different policies for manufacturers and third-party retailers to balance market competition and increase profit. However, it is unclear which hybrid retailing mode (selecting manufacturer or third-party retailer) is more advantageous.

Meanwhile, the product quality distribution strategy has been proposed by platforms in reality. For example, Amazon.com has announced the following standards for brands and/or suppliers selling in the Amazon store: “if any of the Brand’s products are sold by Amazon, the Brand may not also sell those products as a seller in the Amazon store” (Wei and Dong 2022 [1]). For the platforms, under the product quality distribution strategy, some products are only authorized to be sold by the platforms through the reselling channel. Platforms usually choose to sell high-quality products through the reselling channel, and low-quality products are sold in the agency channel. The existing literature has found that the product quality in the reselling mode is higher than that in the agency mode (Xu et al. 2022 [2]), especially when the commission rate is high (Wei and Dong 2022 [1]). Therefore, in this study, we mainly focus on the situation in which the platform sells a high-quality product. Meanwhile, we also investigate the effect of low-quality product sold by the manufacturer on the stakeholders in section **Extension**. For example, for the Lenovo laptop Xiaoxin Air14 sold on JD.com, the high-quality product with a 1 TB SSD is only sold by JD.com through the reselling channel, and the low-quality product with a 512GB SSD is exclusively sold by Lenovo through the agency channel. Moreover, Gree air conditioners are sold on JD.com. Gree provides the high-quality air conditioner Yunjia with first-class efficiency and air purification function for JD.com. The air conditioner Yunjia is sold by JD.com via the reselling channel. At the same time, Gree offers the low-quality air conditioner Yunxuan with third-class energy efficiency in the agency channel. In this case, when the hybrid retailing mode is adopted, the platform makes a retailing structure choice to choose the manufacturer. It forms the competition between the reselling channel (platform) and the agency channel (manufacturer). Furthermore, the product quality distribution strategy proposed by the platform forms competition between product lines with different product qualities. The two types of competition induced by strategy choices simultaneously influence stakeholder decisions and profits. However, the underlying mechanism is still unclear.

These observations motivate the research in this study, which aims to answer the following general question: (1) Should the platform use a product quality distribution strategy? (2) If the product quality distribution strategy is considered concurrently, which sellers, the manufacturer or the third-party retailer, should be chosen by the platform to sell in the hybrid mode? (3) How does the product quality distribution strategy affect the profits and decisions of stakeholders? Based on the aforementioned problems, this paper contributes to the investigation of the platform’s incentive to adopt a product quality distribution strategy by considering the choice of hybrid retailing mode. To answer the above questions, we developed game-theoretic models, and five scenarios were considered: Scenario B, in which the platform sells exclusively through the reselling channel; Scenario RN, under which the third-party retailer sells via the agency channel; Scenario MN, under which the manufacturer sells through the agency channel; Scenario RD, under which the third-party retailer sells the differentiated product and the platform sells the high-quality product through the reselling channel; and Scenario MD, under which the manufacturer sells the differentiated product through the agency channel and the platform sells the high-quality product. Our research sheds light on how platforms choose hybrid retailing modes and determine product distribution strategies based on product category.

Our study’s key findings can be summarized as follows. First, we investigate the impact of the commission rate, product cost, and level of product differentiation on the implementation of a product quality distribution strategy. Our findings indicate that the product quality distribution strategy is only feasible for low product differentiation levels when the third-party retailer sells in the hybrid channel structure because the dual pressures of low pricing power and low-quality product may drive the third-party retailer out of the market. When the manufacturer is chosen, however, the implementation of the product quality distribution strategy will not induce the manufacturer to abandon the agency channel. Second, we investigate how the platform’s equilibrium decision and profitability are affected by the product quality distribution strategy. The results show that under the two types of hybrid channel structures, the product quality distribution strategy can increase the platform’s order quantity. Although the platform sells high-quality products, it can benefit only when a third-party retailer is brought in with a proper commission rate and product differentiation level due to the fierce market competition caused by the product quality distribution strategy. Third, we investigate the impact of the quality differentiation level on stakeholder performance and discover that hybrid channel structures can always improve the manufacturer’s profit. However, once the agency channel is accessible, the manufacturer will not accept product distribution. In addition, two extended scenarios are discussed. We believe that the ability to set different wholesale prices for different sellers benefits the manufacturer. If the platform sells low-quality goods, the manufacturer has a stronger incentive to support the product quality distribution strategy.

The remainder of this paper is organized as follows. Section 2 includes a review of the literature and related studies. In Section 3, we propose the hypotheses and develop the basic model. In Section 4, we establish the models with various hybrid channel structures. In Section 5, the models that are used to implement the product quality distribution strategy are developed, and the optimal joint decision about hybrid mode choice and product quality distribution strategy is analyzed. We conduct extended modeling analyses in Section 6. Section 7 contains conclusions and managerial insights. In Appendices A-C, we elaborate on the analysis for Scenarios RN, RD, and MD, respectively. All proofs are listed in Appendix D in S1 File.

## Literature review

The literature related to our work can be categorized into the following two streams: hybrid retailing mode and product distribution.

Over the past decade, hybrid retailing mode choice has attracted the interest of academics and practitioners. Early studies concentrated primarily on dual-channel supplier chains. For example, Cai et al. (2012) [3] evaluate the joint impact of exclusive channels and revenue sharing on suppliers and retailers in a hybrid duopoly common retailer and exclusive channel model. Cai (2010) [4] investigates the impact of channel structures and channel coordination on the supplier, the retailer, and the entire supply chain using two single-channel and two dual-channel supply chains. Following the emergence of the marketplace, researchers focus more on the transition from a traditional reseller to a hybrid-mode platform from the retailer’s perspective. For example, Mantin et al. (2014) [5] investigate the strategic rationale for a retailer to introduce a 3P marketplace. Hagiu et al. (2015) [6] discuss whether a platform should operate as a marketplace or as a reseller. Abhishek et al. (2016) [7] use a stylized theoretical model to study the timing of using an agency selling format instead of the more conventional reselling format by building models in which two platforms with one of the formats competitively serve a supplier. Zhen and Xu (2022) [8] study which of the sellers (retailer or manufacturer) should use the agency channel provided by a platform. The marketplace mode not only impacts platforms but also affects upstream stakeholders (e.g., manufacturers, suppliers and retailers) in deciding whether to sell via the agency channel. Wang et al. (2018) [9] analyze a manufacturer’s e-channel decision problem in which the manufacturer selects a direct-sales channel or a third-party consignment channel to complement his or her existing physical retail channel. Tian et al. (2018) [10] focus on the impact of the marketplace mode, reseller mode and hybrid mode on the competition between two upstream suppliers. Shen et al. (2019) [11] examine a manufacturer’s decision between platforms with an agency channel and a traditional reseller. Zennyo (2020) [12] studies the competition between two suppliers, each of whom can choose one of two contracts (wholesale or agency) from a platform. Ha et al. (2020) [13] study a situation in which the manufacturer sells the same products through both the agency and reselling channels of the same online retail platform. The recent literature on the hybrid retailing mode has concentrated on several important factors, such as information sharing (Ha et al. 2021 [14], Zhang and Zhang 2020 [15], Wang et al. 2021 [16]), contract design (Siqin et al. 2022 [17]), cooperation among retailers (Wei et al. 2022 [18]) and entry timing (Zhang and Wu 2022 [19]). Unlike previous studies, we investigate how a platform chooses a hybrid retailing mode when the product quality distribution strategy is taken into account.

Our work also relates to the research on product distribution and product quality. Moorthy (1988) [20] studies the impact of consumer preferences and cost on product quality decisions made by firms. Matto (1993) [21] analyzes vertical product differentiation in the context of price and quantity competition. Some studies examine how customer heterogeneity influences product quality (Xu 2009 [22], Shi et al. 2013 [23] and Ha et al. 2016 [24]). Other studies show that product distribution and product quality are also affected by market size (Berry and Waldfogel 2010 [25]), asymmetric information (Chen et al. 2017 [26], Li et al. 2018 [27] and Zhang et al. 2019 [28]), service strategy (Jain and Bala 2018 [29]) and customer demand (Jerath et al. 2017 [30], Zhang et al. 2021 [31]). Furthermore, some researchers investigate the product distribution decision in special circumstances, such as closed-loop supply chains (Orsdemir et al. 2014 [32] and Taleizadeh et al. 2018 [33]) and online and offline markets (Chen et al. 2017 [34]). Considering the competition between manufacturers, Huang et al. (2018) [35] study firms’ decisions about channel structures and product quality levels. Li and Chen (2018) [36] develop game-theoretic models to examine a supply chain in which two manufacturers supply a product in quality-differentiated brands to a common retailer. Following the emergence of hybrid retailing, the main trend has been the manufacturer’s and retailer’s joint decision on channel selection and product quality. For example, Zhang et al. (2019) [37] study the interrelationship between a platform’s contract choice and a manufacturer’s product quality decision. Zhu (2020) [38] studies the impact of the channel structure on the product quality decision made by the manufacturer. Li et al. (2021) [39] characterize the retailer’s optimal encroachment decisions by taking into account the cost–quality trade-off between lower- and higher-quality private labels. Zhang et al. (2022) [40] provide assistance for a dominant supplier manufacturing differentiated products in choosing platforms with different retailing modes. In recent years, researchers have paid more attention to manufacturers’ decisions about product quality distribution strategies for reselling and agency channels (Wei and Dong 2022 [1], Dai et al. 2022 [41] and Luo et al. 2022 [42]). Unlike the aforementioned studies, we find that in reality, platforms have a dominant position in the adoption of product distribution, which may benefit the platforms. Therefore, our study explores product distribution from the perspective of the platform while also taking into account the platform’s decision about the hybrid channel structure.

In the aforementioned literature, game theory is a common method used to study the problems of the hybrid retailing mode and product distribution. Although game-theoretical models are established based on simplified backgrounds, they can not only elaborate the competition and relationship between stakeholders in various retailing structures but also obtain analytical equilibrium solutions to explain stakeholders’ decisions under different scenarios. In particular, Dai et al. (2022) [41] and Luo et al. (2022) [42], the studies that are closest to our work, investigate how to determine the product quality distribution strategy from the perspective of the manufacturer by proposing game theoretical models. Therefore, in this paper, we propose game theoretical models to effectively solve these problems.

## Basic model

### The supply chain

We consider a supply chain that includes a manufacturer (*she*), a platform (*he*), and a third-party retailer. Two kinds of platform retailing channels are considered. The first is the reselling channel, in which the platform purchases products from manufacturers and resells them to consumers (Luo et al. (2022) [42]). The second is the agency channel, in which manufacturers and third-party retailers sell products directly to customers by paying a commission fee with rate *γ* to the platform (Dai et al. (2022) [41]), which is assumed to be exogenous. A platform can choose to provide the agency channel to the manufacturer or third-party retailer.

### Product price

Consumers are heterogeneous in their preferences for product quality. A consumer of type *θ*∈[0,1] is willing to pay *θs* for a product of quality level *s*. This means that the consumer pays more for a product with a higher quality level. Given the product’s price *p*_*H*_, the utility that a consumer of type *θ* buys a product is *θs*−*p*_*H*_. Considering that the manufacturer commits the product quality distribution strategy, another kind of product with a *δ*∈(0,1) fraction of the original product’s quality is provided. Therefore, the differentiated product with low quality provides a utility of *θδs*−*p*_*L*_, where *p*_*L*_ is the differentiated product’s price. If only the original product is provided, a consumer purchases the product only when the utility is positive. Therefore, *θs*−*p*_*H*_≥0, which means that the demand for the original product is $q_{H}=1-\frac{p_{H}}{s}$ and *p*_*H*_ = *s*(1−*q*_*H*_). If the differentiated product is provided at the same time, we have that *p*_*H*_ = *s*(1−*q*_*H*_−*δq*_*L*_) and *p*_*L*_ = *δs*(1−*q*_*H*_−*q*_*L*_), which are applied and validified in Johnson and Myatt (2006) [43], Orsdemir et al. (2014) [32] and Ha et al. (2016) [24].

The unit variable cost of producing a product with quality level *s* is *ks*, where *k*>0 is an exogenous parameter and denotes the cost efficiency. A high value of *k* approximately means that the production cost is high. For the differentiated product, the lower quality occurs at a lower unit production cost, so the unit variable cost is *kδs*. We assume that the selling costs of the platform and the third-party retailer are constant and are normalized to zero.

### Timeline and scenarios

We consider a multistage game with the following sequence of events and decisions: (1) the platform decides whether to provide the agency channel and which of the sellers (the third-party retailer or the manufacturer) can sell the product through the agency channel; meanwhile, the platform decides whether to adopt the product quality distribution strategy; (2) the manufacturer decides on the wholesale price and the order quantity (if selling the product via the agency channel); and (3) the third-party retailer and the platform sequentially decide on the quantities of the products.

According to the aforementioned sequence of decisions, five scenarios are proposed as follows, and practical examples are provided in **Appendix E in S1 File**.

Scenario B: As a simple benchmark, the platform only sells the product via the reselling channel.

In the following scenarios (as shown in **Table 1**), the agency channel is provided by the platform.

**Table 1:** Scenarios based on hybrid channel structure and product quality distribution strategy.

		Product quality distribution	
		Not adopted	Adopted
Agency channel	Third-party retailer	Scenario RN	Scenario RD
	Manufacturer	Scenario MN	Scenario MD

Scenario RN: The third-party retailer sells through the agency channel. The product quality distribution strategy is not proposed by the platform. Therefore, the same product is provided to the platform and the third-party retailer.

Scenario RD: The third-party retailer sells through the agency channel, and the product quality distribution strategy is proposed by the platform. Therefore, the manufacturer provides the low-quality product to the third-party retailer while providing the high-quality product to the platform.

Scenario MN: The manufacturer sells the product via the agency channel, and product distribution is not adopted. The same product is provided to the platform.

Scenario MD: According to the product quality distribution strategy proposed by the platform, the manufacturer sells the low-quality product via the agency channel while providing the high-quality product to the platform.

On summary, in Scenario RN and Scenario RD, the third-party retailer is brought in and sells through the agency channel. In Scenario MN and Scenario MD, the manufacture is brought in and sells through the agency channel. Similarly, in Scenario RN and Scenario MN, the product quality distribution strategy is not adopted. In Scenario RD and Scenario MD, the product quality distribution strategy is adopted, the platform sells the high-quality product, the low-quality product is sold by the third-party retailer or the manufacturer.

Based on the aforementioned sequence of decisions, we analyze the game in backward induction. The firms’ equilibrium decisions are solved first, and then the firms’ profits under different scenarios are computed. We are also curious about the impact on stakeholders if the platform sells low-quality products. Therefore, in the section **Extension**, we discuss two scenarios in which a platform sells low-quality goods under the product quality distribution strategy and a manufacturer sets various wholesale prices in accordance with the retailing channel.

### Scenario B

In Scenario B, only the platform sells through the reselling channel, and the other sellers are not allowed to sell in the platform. Because only the original product is provided, we have the price of the product sold by the platform *p*_*P*_ = *s*(1−*q*_*P*_). First, the manufacturer sets the wholesale price *w*. Then, the platform sets the order quantity *q*_*P*_ to maximize

$$\pi_{P}=(p_{P}-w)q_{P}\;s.t.\;q_{P}\geq 0,$$

which is the same as the function in Ha et al. (2016) [24]. According to the first-order condition (FOC), we can obtain the platform’s best response $q_{p}(w)=\{\begin{array}{ll} \frac{s-w}{2s} & \mathrm{if}\;w<s\\ 0 & \mathrm{otherswise} \end{array}$. Considering the platform’s best response, the manufacturer sets *w* to maximize

$$\pi_{M}=(w-ks)q_{P}.$$

Substituting *q*_*p*_(*w*) into *π*_*M*_, we find that $w^{B}=\frac{(1+k)s}{2}$ when *k*<1 in order that the quantity of product is nonnegative. The quantity of product ordered by the platforms is $q_{P}^{B}=\frac{1-k}{4}$. Superscripts *B* represent the firm’s equilibrium strategies in Scenario B. The platform’s and the manufacturer’s equilibrium profits are given by

$$\pi_{P}^{B}=\frac{{\left(1-k\right)}^{2}s}{16},\pi_{M}^{B}=\frac{{\left(1-k\right)}^{2}s}{8}.$$

## Hybrid channel structure

**Fig 1:**
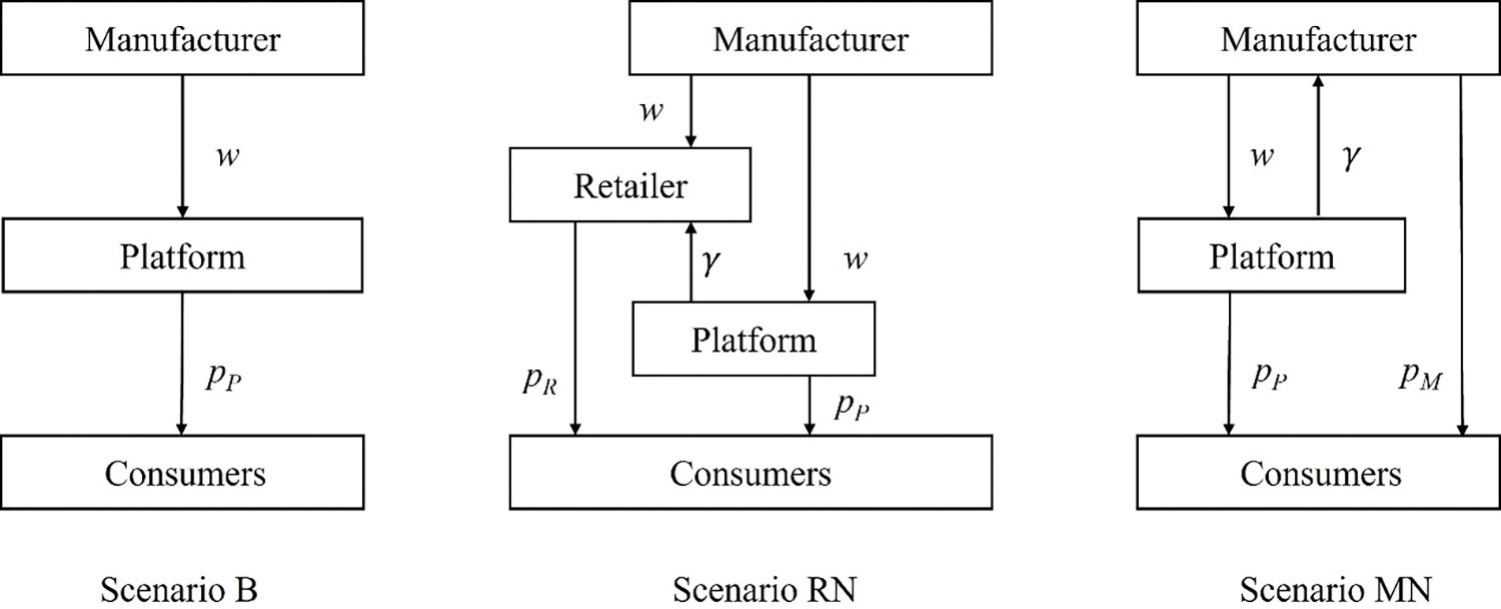
Channel structures in Scenario B, Scenario RN and Scenario MN.

If the platform transitions to the hybrid retailing channel, not only can the platform sell products via the reselling channel, but the third-party retailer or the manufacturer can also sell the product on the platform by paying a commission. Although the platform earns an additional income, at the same time, the platform’s price and order quantity will be impacted by the products sold via the agency channel. Therefore, it is a significant problem for the platform to decide whether to provide the agency channel. If provided, the agency channel is preferred to be opened to the third-party retailer or the manufacturer. In the following, Scenario RN and Scenario MN are compared with Scenario B to answer this question. The channel structures in Scenario B, Scenario RN and Scenario MN are shown in **Fig 1**.

### Scenario RN

In Scenario RN, the third-party retailer sells products via the agency channel. A proportion *γ* of the revenue is paid to the platform. Meanwhile, the platform does not propose the product quality distribution strategy, so the manufacturer provides the same product to the platform and the third-party retailer. Therefore, we have the price of the product *p*_*P*_ = *p*_*R*_ = *s*(1−*q*_*P*_−*q*_*R*_). Observing the third-party retailer’s order, the platform decides *q*_*P*_ to maximize his profit

$$\pi_{P}=(p_{p}-w)q_{p}+\gamma p_{R}q_{R}\;s.t.\;q_{P}\geq 0.$$

The platform’s profit includes two parts: (*p*_*p*_−*w*)*q*_*p*_ is the profit from the reselling channel, and *γp*_*R*_*q*_*R*_ is the profit from the agency channel. From the FOC, we can obtain the platform’s best response as follows:

$$q_{P}=\{\begin{array}{ll} \frac{s-w-(1+\gamma )q_{R}s}{2s} & \mathrm{if}\;q_{R}\leq \frac{s-w}{(1+\gamma )s},\\ 0 & \mathrm{otherwise}. \end{array}$$

If the third-party retailer places a large order ($q_{R}>\frac{s-w}{(1+\gamma )s}$), the platform will not sell the product via the reselling channel (i.e., *q*_*P*_ = 0) to maximize his profit. Given the wholesale price *w*, the third-party retailer decides *q*_*R*_ to maximize profit

$$\pi_{R}(q_{R})=(1-\gamma )p_{R}q_{R}-wq_{R}\;s.t.\;q_{R}\geq 0.$$

The third-party retailer’s profit also contains two parts, including the revenue from selling via the agency channel and the production cost. The aforementioned profit functions are the same as those in Dai et al. (2022) [41]. From the FOC, we can derive the third-party retailer’s best response:

(i) if $\frac{1}{3}<\gamma <1$,

$$q_{R}(w)=\{\begin{array}{ll} 0 & \mathrm{if}\;w>\frac{(1-\gamma )s}{1+\gamma },\\ \frac{(1-\gamma )s-(1+\gamma )w}{2{\left(1-\gamma \right)}^{2}s} & \mathrm{if}\;\frac{(1-\gamma )(1-3\gamma )s}{\gamma^{2}-6\gamma +1}<w\leq \frac{(1-\gamma )s}{1+\gamma },\\ \frac{s-w}{(1+\gamma )s} & \mathrm{if}\;\frac{{\left(1-\gamma \right)}^{2}s}{1-3\gamma }<w\leq \frac{(1-\gamma )(1-3\gamma )s}{\gamma^{2}-6\gamma +1},\\ \frac{(1-\gamma )s-w}{2(1-\gamma )s} & \mathrm{if}\;w\leq \frac{{\left(1-\gamma \right)}^{2}s}{1-3\gamma }. \end{array}$$

(ii) if $3-2\sqrt{2}<\gamma <\frac{1}{3}$,

$$q_{R}(w)=\{\begin{array}{ll} 0 & \mathrm{if}\;w\geq \frac{(1-\gamma )s}{1+\gamma },\\ \frac{(1-\gamma )s-(1+\gamma )w}{2{\left(1-\gamma \right)}^{2}s} & \mathrm{if}\;\frac{(1-\gamma )(1-3\gamma )s}{\gamma^{2}-6\gamma +1}<w<\frac{(1-\gamma )s}{1+\gamma },\\ \frac{s-w}{(1+\gamma )s} & \mathrm{if}\;w\leq \frac{(1-\gamma )(1-3\gamma )s}{\gamma^{2}-6\gamma +1}. \end{array}$$

(iii) if $0<\gamma <3-2\sqrt{2}$,

$$q_{R}(w)=\{\begin{array}{ll} 0 & \mathrm{if}\;w\geq \frac{(1-\gamma )s}{1+\gamma },\\ \frac{(1-\gamma )s-(1+\gamma )w}{2{\left(1-\gamma \right)}^{2}s} & \mathrm{if}\;w<\frac{(1-\gamma )s}{1+\gamma }. \end{array}$$

When the wholesale price is high ($w>\frac{(1-\gamma )s}{1+\gamma }$), the third-party retailer will abandon the order regardless of the commission rate. Meanwhile, the platform will also sell nothing via the reselling channel under the significantly high wholesale price. If the commission rate is high ($\frac{1}{3}<\gamma <1$), the platform will gain the most share of profit via the agency channel. Therefore, the third-party retailer is more sensitive to the wholesale price. When the wholesale price is low, the third-party retailer will place a large order ($q_{R}(w)=\frac{s-w}{(1+\gamma )s}$ or $q_{R}(w)=\frac{(1-\gamma )s-w}{2(1-\gamma )s}$) to entice the platform not to sell via the reselling channel (*q*_*P*_ = 0). As a result, the manufacturer only obtains profit via the agency channel. It is worth noting that the order $q_{R}(w)=\frac{s-w}{(1+\gamma )s}$ causes the platform to decide as if there is no reselling channel, but the order $q_{R}(w)=\frac{(1-\gamma )s-w}{2(1-\gamma )s}$ makes the platform give up the reselling channel. When the wholesale price is relatively high, the third-party retailer will place a small order, forcing the platform to sell via the reselling channel to obtain more profit. If the commission rate is median ($3-2\sqrt{2}<\gamma <\frac{1}{3}$), the third-party retailer’s order decision will not compel the platform to abandon the reselling channel. When $0<\gamma <3-2\sqrt{2}$, if the order quantity is large enough to force the platform to refuse the reselling channel, the product price will decrease, as will the third-party retailer’s profit. As a result, the third-party retailer will select a low order quantity, and the two channels will coexist.

Anticipating *q*_*R*_(*w*) and *q*_*P*_(*w*), the manufacturer can set *w* to maximize her profit

$$\pi_{M}=(w-ks)(q_{R}+q_{P}).$$

We can derive the equilibrium wholesale price:

$$w^{RN}=\{\begin{array}{ll} s & \mathrm{if}\;0<\gamma <1,k>1,\\ \frac{(1+k)s}{2} & \mathrm{if}\;\frac{3}{7}<\gamma <1,0<k<1,\\ & \mathrm{or}\;0<\gamma <\frac{3}{7},k^{RN}<k<1,\\ \frac{(3(1-\gamma )+(3-\gamma )k)s}{2(3-\gamma )} & \mathrm{if}\;0<\gamma \leq \frac{3}{7},0<k<k^{RN}, \end{array}$$

where $k^{RN}=\frac{\left(1-8\gamma \sqrt{\frac{1}{8(\gamma -1)(\gamma -3)}})(1-\gamma \right)}{\gamma +1}$. Superscripts *RN* represent the firm’s equilibrium strategies and relative parameters in Scenario RN. Given the quality of the product, the cost of the product is proportional to the parameter *k*. Therefore, a higher value of *k* indicates that the product is more costly. Only when the cost level is low and the commission rate is low enough ($0<\gamma \leq \frac{3}{7},0<k<k^{RN}$) will the manufacturer support the existence of the dual channels. The higher commission rate reduces the third-party retailer’s profit. Furthermore, the platform’s profit decreases due to the higher cost of the product, which causes the product’s price to increase even further. As a result, as the commission rate or cost of the product rises, the manufacturer chooses a high wholesale price to maintain profit, causing the third-party retailer to leave and the platform to expand his sales to occupy the market. When the cost of the product exceeds the critical point (*k*>1), the manufacturer chooses a larger wholesale price, making the profit nonnegative, even if both the platform and the third-party retailer cannot sell anything, as in Scenario B. Plugging *w*^*RN*^ into the firms’ order quantities, we can have their equilibrium quantities:

$$q_{P}^{RN}=\{\begin{array}{ll} 0 & \mathrm{if}\;0<\gamma <1,k>1,\\ \frac{1-k}{4} & \mathrm{if}\;\frac{3}{7}<\gamma <1,0<k<1,\\ & \mathrm{or}\;0<\gamma <\frac{3}{7},k^{RN}<k<1,\\ \frac{(3\gamma^{2}-2\gamma +3)(1-\gamma )+(3-\gamma )(\gamma^{2}-6\gamma +1)k}{8{\left(1-\gamma \right)}^{2}(3-\gamma )}\\ & \mathrm{if}\;0<\gamma \leq \frac{3}{7},0<k<k^{RN}, \end{array}$$

$$q_{R}^{RN}=\{\begin{array}{ll} 0 & \mathrm{if}\;\frac{3}{7}<\gamma <1,0<k<1,\\ & \mathrm{or}\;0<\gamma <\frac{3}{7},k^{RN}<k<1,\\ \frac{(1-\gamma )(3-5\gamma )-(3-\gamma )(1+\gamma )k}{4{\left(1-\gamma \right)}^{2}(3-\gamma )} & \mathrm{if}\;0<\gamma \leq \frac{3}{7},0<k<k^{RN}. \end{array}$$

The firms’ equilibrium profits are given in Appendix A in S1 File.

### Scenario MN

In Scenario MN, the manufacturer sells products via the agency channel. A proportion *γ* of the revenue is paid to the platform. Meanwhile, because the platform does not propose a product quality distribution strategy, the manufacturer sells the same product as the platform. Therefore, we have the price of the product *p*_*P*_ = *p*_*M*_ = *s*(1−*q*_*P*_−*q*_*M*_). Observing the platform’s wholesale price and order quantity, the platform sets *q*_*P*_ to maximize his profit

$$\pi_{P}=(p_{p}-w)q_{p}+\gamma p_{M}q_{M}\;s.t.\;q_{P}\geq 0,$$

which is also used in Luo et al. (2022) [42]. From the FOC, we can obtain the platform’s best response as follows:

$$q_{P}=\{\begin{array}{ll} \frac{s-w-(1+\gamma )q_{M}s}{2s} & \mathrm{if}\;1-(1+\gamma )q_{M}-\frac{w}{s}\geq 0,\\ 0 & \mathrm{otherwise}. \end{array}$$

The platform decides on the order quantity by comprehensively considering the manufacturer’s wholesale price and order quantity. If the platform has a large order or the wholesale price is too high, the platform will not sell the product via the reselling channel. The manufacturer sets *q*_*M*_ and *w* to maximize the profit

$$\pi_{M}(q_{M},w)=(1-\gamma )p_{M}q_{M}+wq_{P}-ks(q_{M}+q_{P})\;s.t.\;q_{M}\geq 0.$$

The manufacturer’s profit also has three parts, including the revenue from selling via the agency channel, the revenue from providing the product to the platform, and the production cost. From the FOC, we can derive the manufacturer’s equilibrium wholesale price and order quantity:

$$w^{MN}=\{\begin{array}{ll} s & \mathrm{if}\;0<\gamma <1,k>1,\\ \frac{1+k}{2}s & \mathrm{if}\;0<\gamma <\frac{1}{2},k^{MN}<k<1,\\ & \mathrm{or}\;\frac{1}{2}<\gamma <1,0<k<1,\\ \frac{\left(1-\gamma )(k-2\gamma +1\right)}{2(1-2\gamma )}s & \mathrm{if}\;0<\gamma <\frac{1}{2},0<k<k^{MN}, \end{array}$$

$$q_{M}^{MN}=\{\begin{array}{ll} 0 & \mathrm{if}\;0<\gamma <1,k>1,\\ & \mathrm{or}\;0<\gamma <\frac{1}{2},k^{MN}<k<1,\\ & \mathrm{or}\;\frac{1}{2}<\gamma <1,0<k<1,\\ \frac{1-2\gamma -k}{2(1-2\gamma )} & \mathrm{if}\;0<\gamma <\frac{1}{2},0<k<k^{MN}. \end{array}$$

where *k*^*MN*^ = 1−2*γ*. Superscripts *MN* represent the firm’s equilibrium strategies and relative parameters in Scenario MN.

Substituting *w*^*MN*^ and $q_{M}^{MN}$ into *q*_*P*_(*w*,*q*_*M*_), we can have the platform’s equilibrium order quantity

$$q_{P}^{MN}=\{\begin{array}{ll} 0 & \mathrm{if}\;0<\gamma <1,k>1,\\ \frac{1-k}{4} & \mathrm{if}\;0<\gamma <\frac{1}{2},k^{MN}<k<1,\\ & \mathrm{or}\;\frac{1}{2}<\gamma <1,0<k<1,\\ \frac{\gamma k}{2(1-2\gamma )} & \mathrm{if}\;0<\gamma <\frac{1}{2},0<k<k^{MN}. \end{array}$$

Plugging *w*^*MN*^, $q_{M}^{MN}$ and $q_{P}^{MN}$ into the firm’s profit functions, we have

$$\pi_{P}^{MN}=\{\begin{array}{ll} 0 & \mathrm{if}\;0<\gamma <1,k>1,\\ \frac{{\left(1-k\right)}^{2}}{16}s & \mathrm{if}\;0<\gamma <\frac{1}{2},k^{MN}<k<1,\\ & \mathrm{or}\;\frac{1}{2}<\gamma <1,0<k<1,\\ \frac{\gamma ({\left(1-2\gamma \right)}^{2}-(1-\gamma )k^{2})}{4{\left(1-2\gamma \right)}^{2}}s & \mathrm{if}\;0<\gamma <\frac{1}{2},0<k<k^{MN}, \end{array}$$

$$\pi_{M}^{MN}=\{\begin{array}{ll} 0 & \mathrm{if}\;0<\gamma <1,k>1,\\ \frac{{\left(1-k\right)}^{2}}{8}s & \mathrm{if}\;0<\gamma <\frac{1}{2},k^{MN}<k<1,\\ & \mathrm{or}\;\frac{1}{2}<\gamma <1,0<k<1,\\ \frac{\left((1-\gamma -2k)(1-2\gamma )+(1-\gamma )k^{2}\right)}{4(1-2\gamma )}s & \mathrm{if}\;0<\gamma <\frac{1}{2},0<k<k^{MN}. \end{array}$$

The equilibrium outcomes under the above scenarios show that if the commission rate exceeds a certain threshold (i.e., $\frac{3}{7}\approx 0.429$ in Scenario RN, $\frac{1}{2}$ in Scenario MN), the third-party retailer or the manufacturer will refuse to participate in agency selling. These findings are consistent with the actual situation. For example, Amazon.com sets the highest commission rate as 45% for its devices and accessories (“Selling on Amazon Fee Schedule”). In most situations, platforms such as JD.com and Amazon.com set a commission rate of no more than 50% for the products sold by agency sellers.

### Compare and analysis

#### The platform’s decision on the agency channel

To analyze the platform’s decision on the selection of the third-party retailer, we compare the platform’s equilibrium order quantity and profit in Scenarios B and RN.

#### Lemma 1

- *When the commission rate is high or the product is costly, the selection of the third-party retailer has no effect on the platform’s order quantity because the third-party retailer will not sell the product through the platform. Specifically, if $\frac{3}{7}<\gamma <1$ or k*^*RN*^<*k*<1, $q_{R}^{RN}=0$ and $q_{P}^{B}=q_{P}^{RN}$.
- *When the commission rate is appropriate ($0<\gamma <\frac{3}{7}$), if k*_1_<*k*<*k*^*RN*^*, the platform’s order quantity will increase due to the selection of the third-party retailer ($q_{P}^{B}<q_{P}^{RN}$); otherwise, if* 0<*k*<*k*_1_, *the platform will sell fewer products through the reselling channel* ($q_{P}^{B}>q_{P}^{RN}$).

Inferentially, we might anticipate that the addition of the third-party retailer would result in a lower order quantity for the platform in Scenario RN than in Scenario B. However, we have discovered that this is not always the case. Part (a) of **Lemma 1** shows that the third-party retailer will decline the platform’s invitation due to the high commission rate or the high cost of the product. In this case, the third-party retailer’s revenue cannot cover the payment, so the best option is to close the agency channel.

Due to the fierce competition in the downstream market, it is commonly believed that the platform will sell fewer units than in Scenario B. However, the Part (b) of **Lemma 1** demonstrates that it is not always true. Specifically, if the production cost is high (i.e., *k*_1_<*k*<*k*^*RN*^), even if the selection of the third-party retailer causes the price of the product to fall and the total quantity to rise, the high wholesale price caused by the cost limits the third-party retailer’s order quantity. As a result, even the competitor sells in the same market, the platform’s order quantity still conversely increases. When the cost is low (i.e., 0<*k*<*k*_1_), the third-party retailer earns a higher profit margin from the agency channel, which encourages to order more quantity. Meanwhile, for the platform, the best strategy is to avoid ordering more products, which intensifies the competition and lower the price. So, the platform chooses to shrink his market share and highly relies on the profit from the agency channel. Therefore, the platform sells fewer products in Scenario RN than Scenario B.

In order to study the impact of third-party retailer on the total retail quantities in the market, the following lemma is provided.

#### Lemma 2

*When the third-party retailer sells the product via the agency channel, the total number of products sold through the platform increases than before. Specifically, $0<\gamma \leq \frac{3}{7},0<k<k^{RN}$, we always have $q_{P}^{B}<Q^{RN}$, where Q* = *q*_*R*_+*q*_*P*_.

According to Lemma 1, we can find that when the cost is moderative (*k*_1_<*k*<*k*^*RN*^), the fierce competition caused by the third-party retailer forces the platform to maintain his profit by increasing sales via the reselling channel. It is clearly that the total quantity in Scenario RN is larger than that in Scenario B. In addition, When the cost is low (0<*k*<*k*_1_), the platform observes a higher profit margin from the third-party retailer. Therefore, in order to balance and maximize the profits earned from the agency channel and reselling channel, the platform limitedly shrinks the order quantity. The quantity of the declining order is still less than the third-party retailer’s new order quantity. Therefore, in summary, the total quantity in Scenario RN is always larger than that in Scenario B.

The previous lemmas explain how the selection of the third-party retailer affects the platform’s order. Based on Scenario MN, the following lemmas will describe the impact of the manufacturer’s channel expansion.

#### Lemma 3

*(a)For the platform, the order quantity will decrease, when the manufacturer sells the product via the agency channel. Specifically, $q_{P}^{B}>q_{P}^{MN}$, when $0<\gamma <\frac{1}{2},0<k<k^{MN}$. (b) when the cost of the product is low (*0<*k*<*k*_2_*), the platform’s order quantity is less than the manufacturer’s ($q_{P}^{MN}<q_{M}^{MN}$); otherwise, the platform sells more product than the manufacturer.*

In comparison to **Lemma 1**, we find that the selection of the manufacturer has a significantly negative impact on the platform’s order quantity. Because of the potential competition from the agency channel, the manufacturer has a natural advantage over the platform, forcing the platform to reduce order quantity in order to respond to the increase in wholesale price. Furthermore, **Lemma 3(b)** demonstrates that a lower production cost (0<*k*<*k*_2_) allows the manufacturer to earn a higher profit margin and dominate the market. In the interim, the platform must rely on the agency channel and select a low order quantity. However, even when production costs are relatively high, the platform can maintain a market share advantage because less efficient production reduces the manufacturer’s profit margin in the agency channel.

The following lemma illustrates the effect of the manufacturer on the total order quantity.

#### Lemma 4

For the platform, when the manufacturer sells the product via the agency channel, the total number of products sold through the platform increases. Specifically, $q_{P}^{B}<Q^{MN}$, when $0<\gamma <\frac{1}{2},0<k<k^{MN}$.

Similar to **Lemma 3**, when the manufacturer begins to sell products through a hybrid platform, the total order quantity increases. Under the influence of competition, regardless of changes of the platform’s order quantity, the sellers using the agency channel always fill the market gap, consequently reducing the product’s price and increasing the potential demand.

To study the platform’s decision on the channel structure, the following proposition is provided.

#### Proposition 1

- *The platform chooses that the third-party retailer sells the product via the agency channel* ($\pi_{P}^{B}<\pi_{P}^{RN}$), *when* $\gamma_{1}<\gamma <\frac{3}{7},0<k<k^{RN}$ *or* $0<\gamma <\gamma_{1},k_{3}<k<k^{RN}$. *Otherwise, when* 0<*γ*<*γ*_1_,0<*k*<*k*_3_, *the platform sells solely through the reselling channel*.
- *The platform chooses the manufacturer to sell the product via the agency channel ($\pi_{P}^{B}<\pi_{P}^{MN}$), when* $\frac{1}{4}<\gamma <\frac{1}{2},0<k<k^{MN}$ *or $0<\gamma <\frac{1}{4},k_{4}<k<k^{RN}$. Otherwise, the platform sells only through the reselling channel.*
- *Compared with the introduction of the third-party retailer, the platform prefers to invite the manufacturer* ($\pi_{P}^{MN}=\max(\pi_{P}^{B},\pi_{P}^{RN},\pi_{P}^{MN})$), when $\frac{1}{3}<\gamma <\gamma_{2},0<k<k_{5}$ or $\gamma_{2}<\gamma <\frac{3}{7},0<k<k^{RN}$. Otherwise, the introduction of the third-party retailer is the best choice ($\pi_{P}^{RN}=\max(\pi_{P}^{B},\pi_{P}^{RN},\pi_{P}^{MN})$, *when* 0<*γ*<*γ*_1_,*k*_3_<*k*<*k*^*RN*^ *or* $\gamma_{1}<\gamma <\frac{1}{3},0<k<k^{RN}$ or $\frac{1}{3}<\gamma <\gamma_{2},k_{5}<k<k^{RN}$.

There are two questions regarding the platform’s channel structure: whether the agency channel should be added; (2) if the agency channel is added, which third-party firms should be chosen. With the third-party retailer chosen, **Proposition 1(a)** shows that if the commission rate is intermediate ($\gamma_{1}<\gamma <\frac{3}{7}$), the platform can use the agency channel to earn more profit than Scenario B by charging a generally high commission fee proportional to the third-party retailer’s revenue. If the commission rate is low (0<*γ*<*γ*_1_), the relatively high cost (*k*_3_<*k*<*k*^*RN*^) limits the third-party retailer’s sales while increasing the platform’s order quantity (discussed in **Lemma 1**). Thus, in this case, the platform’s profit is significantly higher than in Scenario B, and he chooses to invite the third-party retailer. **Proposition 1(b)** discusses the results on the platform’s profit in Scenario MN. Similarly, a higher commission rate makes the platform better off. And when the commission rate is low, the excess profit depends on the less efficient production cost (i.e., *k*_4_<*k*<*k*^*RN*^). In comparison Scenario RN, we can find that if the platform wants to choose the manufacturer to get more profit, the lower threshold of the commission rate condition is higher (i.e., $\frac{1}{4}>\gamma_{1}$). It is because that the manufacturer can sell her product via both the agency and reselling channels, which means she has more bargaining power than the third-party retailer. **Proposition 1(c)** illustrates the platform’s channel structure decision when considering both the third-party retailer and the manufacturer’s introductions. The result is more complicated. Generally speaking, the platform is more likely to choose the manufacturer, when the commission rate is relatively high. Because, when compared to the third-party retailer, even the high commission rate reduces the manufacturer’s profit margin from the agency channel, she can still maintain the profit by adjusting the wholesale price and keep the amount of sales. As a result, when the manufacturer sells through the agency channel, the platform benefits from the higher commission rate. Furthermore, the intermediate commission rate with a proper production cost more can likely enable the platform to choose the introduction of the third-party retailer. The intermediate commission rate may alleviate the third-party retailer’s cost concerns, resulting in higher sales than the manufacturer’s sales. Meanwhile, the platform also sells more in Scenario RN than in Scenario MN. Therefore, in Scenario RN, the platform can profit more in this situation. It is worth noting that when commission rate is low (0<*γ*<*γ*_1_.), the platform’s choice is relative with the production cost. If the cost parameter level is much less than a threshold (*k*<*k*_3_), the platform’s profit can be deteriorated by the agency channel, and he sells solely via the reselling channel. To some extent, **Proposition 1** can explain the reason why the platforms, such as JD.com and Amazon.com, want to choose the third-party firms and provide the agency channel.

### The impact of the agency channel on the manufacturer

#### Lemma 5

*No matter whether the product is sold through the agency channel by a third-party retailer or the manufacturer*, *the manufacturer’s wholesale price decreases*. *That is*, *w*^*B*^>*w*^*RN*^*, if $0<\gamma <\frac{3}{7}$ and* 0<*k*<*k*^*RN*^ *(Scenario RN);*.*w*^*B*^>*w*^*MN*^*, if* $0<\gamma <\frac{1}{2}$ *and* 0<*k*<*k*^*MN*^ *(Scenario MN). Thus, $w_{}^{B}=\max(w_{}^{B},w_{}^{RN},w_{}^{MN})$, if $0<\gamma <\frac{3}{7}$ and* 0<*k*<*k*^*RN*^.

In Scenario RN, when the third-party retailer sells through the agency channel, the fierce competition lowers the price of the product. As a result, in order for the manufacturer to maximize her profit, it is preferable to encourage the third-party retailer to stay in the market. The third-party retailer’s profit margin is still positive, thus she is forced to reduce the wholesale price. Furthermore, if the manufacturer can sell directly in the market (Scenario MN), we think that a higher wholesale price is preferable for the manufacturer to increase profit from the reselling channel. However, the Lemma 5 demonstrates that the manufacturer continues to lower the wholesale price to offset the price change and maintain the platform’s profit margin. Only in this manner can the manufacturer minimize the impact of the introduction on the profit from the reselling channel and maximize her profit. In summary, in Scenario B, the manufacturer sets the highest wholesale price compared to the other scenarios.

The following proposition explicates the impact of the agency channel on the manufacturer’s profit.

#### Proposition 2

*(a) The manufacturer will be better off, when the agency channel is provided by the manufacturer. That is, $\pi_{M}^{B}<\pi_{M}^{RN}$, if $0<\gamma <\frac{3}{7}$ and* 0<*k*<*k*^*RN*^*; $\pi_{M}^{B}<\pi_{M}^{MN}$, if $0<\gamma <\frac{1}{2}$ and* 0<*k*<*k*^*MN*^*. (b) Meanwhile, the manufacturer always prefers to directly sell via the agency channel than selling to the third-party retailer or only to the platform, which means that $\pi_{M}^{MN}=\max(\pi_{M}^{B},\pi_{M}^{RN},\pi_{M}^{MN})$, if $0<\gamma <\frac{3}{7}$ and* 0<*k*<*k*^*RN*^.

When the platform changes from the pure reselling channel to the hybrid channel, the manufacturer’s reaction is critical. Proposition 2 (a) demonstrates that, when compared to the pure reselling format (Scenario B), the hybrid format, which **utilizes** both the agency channel and reselling, always benefits the manufacturer. In Scenario RN, the fact that the total order quantity increases, as demonstrated in Lemma 2, effectively benefits the manufacturer, even if the wholesale price decreases slightly. In Scenarios MN, the manufacturer can sell directly to consumers. Although the decline in platform sales reduces her reselling channel profit, the manufacturer is still better off because the profit from the agency channel can completely offset the loss. As a result, the manufacturer is always incentivized to support the platform’s adoption of hybrid channel. When we compare the manufacturer’s profit under the three scenarios, we discover that the most profitable strategy for the manufacturer is to sell directly through the agency channel, which is intuitive.

## Product quality distribution strategy

When third-party firms can sell the product through the agency channel, the platform’s market share will decline. Product distribution is one of the most effective ways for a platform to maintain its priorities. This implies that the manufacturer must provide a differentiated product of varying quality to the sellers in the agency channel. However, it is still unclear whether the product quality distribution strategy has a positive impact on stakeholders. Therefore, in this section, we will discuss the abovementioned issues. Based on Scenarios MN and RN, we assume that the low-quality product is sold through the agency channel by the third-party seller (the third-party retailer in Scenario MD or the manufacturer in Scenario RN). In the section **Extension**, we will also examine the scenario in which the platform sells the differentiated (low-quality) product. To capture the consumers’ preference for the low-quality product, we assume that the quality of the differentiated product is a *δ* fraction of the high-quality product where *δ*∈(0,1), which is similar to the assumption in Orsdemir et al. (2014) [32].

### Scenario RD

In Scenario RD, the platform adopts the product quality distribution strategy. Therefore, the manufacturer provides the differentiated low-quality product to the third-party retailer. The product sold by the platform is unchanged. According to the assumptions in **Section 3**, the prices of the products sold by the platform and by the third-party retailer are *p*_*P*_ = *s*(1−*q*_*P*_−*δq*_*R*_) and *p*_*R*_ = *δs*(1−*q*_*P*_−*q*_*R*_), respectively. In Scenario RD, due to the simplicity of model deduction and outcome analysis, we hypothesize that the wholesale prices of the two kinds of products are the same. In **Section 6**, we will explain the impact of different wholesale prices.

The decision sequence is the same as in Scenario RN, and the profit functions of the platform and third-party retailer remain unchanged. Because of product distribution, the manufacturer determines the wholesale price based on profit, as shown below.


$$\pi_{M}=(w-ks)q_{R}+(w-k\delta s)q_{p}.$$

We can derive the equilibrium wholesale price and the firms’ equilibrium order quantities as follows:

$$w^{RD}=\{\begin{array}{ll} s & \mathrm{if}\;0<\gamma <1,0<\delta \leq 1,k>1,\\ \frac{(1+k)s}{2} & \mathrm{if}\;0<\gamma <1,0<\delta \leq 1,k^{RD}<k<1,\\ & \mathrm{or}\;\frac{3}{7}<\gamma <1,0<\delta \leq 1,0<k<k^{RD},\\ & \mathrm{or}\;0<\gamma <\frac{3}{7},0<\delta <\frac{4}{7(1-\gamma )},0<k<k^{RD},\\ \frac{(3(\delta +\delta \gamma -2)+k(3\delta +\delta \gamma -6))(1-\gamma )\delta s}{2(\delta -\delta \gamma +2)(\delta +\delta \gamma -2)}\\ & \mathrm{if}\;0<\gamma <\frac{3}{7},\frac{4}{7(1-\gamma )}<\delta \leq 1,0<k<k^{RD}, \end{array}$$

$$q_{P}^{RD}=\{\begin{array}{ll} 0 & \mathrm{if}\;0<\gamma <1,0<\delta \leq 1,k>1,\\ \frac{1-k}{4} & \mathrm{if}\;0<\gamma <1,0<\delta \leq 1,k^{RD}<k<1,\\ & \mathrm{or}\;\frac{3}{7}<\gamma <1,0<\delta \leq 1,0<k<k^{RD},\\ & \mathrm{or}\;0<\gamma <\frac{3}{7},0<\delta <\frac{4}{7(1-\gamma )},0<k<k^{RD},\\ \frac{\delta k(3\delta +\delta \gamma -6)(\delta \gamma^{2}-6\gamma -\delta +2)-(\delta +\delta \gamma -2)(3\delta^{2}\gamma^{2}-3\delta^{2}-2\delta \gamma -10\delta +16)}{8{\left(\delta +\delta \gamma -2\right)}^{2}(\delta -\delta \gamma +2)}\\ & \mathrm{if}\;0<\gamma <\frac{3}{7},\frac{4}{7(1-\gamma )}<\delta \leq 1,0<k<k^{RD}, \end{array}$$

$$q_{R}^{RD}=\{\begin{array}{ll} 0 & \mathrm{if}\;0<\gamma <1,0<\delta \leq 1,k>k^{RD},\\ & \mathrm{or}\;\frac{3}{7}<\gamma <1,0<\delta \leq 1,0<k<k^{RD},\\ & \mathrm{or}\;0<\gamma <\frac{3}{7},0<\delta <\frac{4}{7(1-\gamma )},0<k<k^{RD},\\ \frac{k(3\delta +\delta \gamma -6)(2-\delta +\delta \gamma )+(2-\delta -\delta \gamma )(5\delta -5\delta \gamma -2)}{4{\left(\delta +\delta \gamma -2\right)}^{2}{\left(\delta -\delta \gamma +2\right)}^{2}}\\ & \mathrm{if}\;0<\gamma <\frac{3}{7},\frac{4}{7(1-\gamma )}<\delta \leq 1,0<k<k^{RD}. \end{array}$$

The firms’ equilibrium profits and detailed analysis are in Appendix B in S1 File.

### Scenario MD

In Scenario MD, the manufacturer provides a differentiated product according to the platform’s request. We assume that the differentiated (low-quality) product is sold by the manufacturer, which is similar to the assumption in Wei and Dong (2022) [1]. Therefore, the prices of the products sold by the platform and by the manufacturer are *p*_*P*_ = *s*(1−*q*_*P*_−*δq*_*M*_) and *p*_*M*_ = *δs*(1−*q*_*P*_−*q*_*M*_), respectively. The sequence of decisions is the same as in Scenario MN. Because of product distribution, the manufacturer determines the wholesale price and order quantity based on profit, as shown below.


$$\pi_{M}(q_{M},w)=((1-\gamma )p_{M}-k\delta s)q_{M}+(w-ks)q_{P}\;s.t.\;q_{P}\geq 0.$$

We can derive the equilibrium wholesale price and the firm’s equilibrium order quantities as follows:

$$w^{MD}=\{\begin{array}{ll} s & \mathrm{if}\;0<\gamma <1,0<\delta \leq 1,k>1,\\ \frac{1+k}{2}s & \mathrm{if}\;0<\gamma <\frac{1}{2},0<\delta \leq 1,k^{MD}<k<1,\\ & \mathrm{or}\;\frac{1}{2}<\gamma <1,0<\delta \leq 1,0<k<1,\\ \frac{\left(1-\gamma )(2-\delta -2\delta \gamma +(2-\delta )k\right)}{2(2-\delta -2\gamma )}s\\ & \mathrm{if}\;0<\gamma <\frac{1}{2},0<\delta \leq 1,0<k<k^{MD}, \end{array}$$

$$q_{M}^{MD}=\{\begin{array}{ll} 0 & \mathrm{if}\;0<\gamma <1,0<\delta \leq 1,k>1,\\ & \mathrm{or}\;0<\gamma <\frac{1}{2},0<\delta \leq 1,k^{MD}<k<1,\\ & \mathrm{or}\;\frac{1}{2}<\gamma <1,0<\delta \leq 1,0<k<1,\\ \frac{1-2\gamma -k}{2(2-\delta -2\gamma )} & \mathrm{if}\;0<\gamma <\frac{1}{2},0<\delta \leq 1,0<k<k^{MD}, \end{array}$$

$$q_{P}^{MD}=\{\begin{array}{ll} 0 & \mathrm{if}\;0<\gamma <1,0<\delta \leq 1,k>1,\\ \frac{1-k}{4} & \mathrm{if}\;0<\gamma <\frac{1}{2},0<\delta \leq 1,k^{MD}<k<1,\\ & \mathrm{or}\;\frac{1}{2}<\gamma <1,0<\delta \leq 1,0<k<1,\\ \frac{(1-\delta )(1-\gamma -k)+\gamma k}{2(2-\delta -2\gamma )}\\ & \mathrm{if}\;0<\gamma <\frac{1}{2},0<\delta \leq 1,0<k<k^{MD}. \end{array})$$

where *k*^*MD*^ = *k*^*MN*^ = 1−*γ*.

The firms’ equilibrium profits and detailed analysis are presented in Appendix C in S1 File.

### Comparison and analysis

#### The impact of the product quality distribution strategy on the platform

Intuitively, we find that when *δ* = 1, the firms’ equilibrium strategies in Scenario RD and Scenario MD are the same as those in Scenario RN and Scenario MN, respectively. To analyze the impact of the product quality distribution strategy, we provide the following lemmas and propositions. First, we focus on the impact on the platform.

#### Lemma 4

*When the third-party retailer or the manufacturer sells the product via the agency channel, the product quality distribution strategy can inspire the platform’s order quantity. That is, $q_{P}^{RD}>q_{P}^{RN}$, if $0<\gamma <\frac{3}{7}$, $\frac{4}{7(1-\gamma )}<\delta \leq 1$ and* 0<*k*<*k*^*RD*^, $q_{P}^{MD}>q_{P}^{MN}$, if $0<\gamma <\frac{1}{2}$, 0<*δ*≤1 *and* 0<*k*<*k*^*MD*^.

When the manufacturer sells the differentiated product through the agency channel, the platform’s order quantity increases. This is because the manufacturer’s low-quality product reduces the consumer’s utility in consuming these products. As a result, consumers will purchase fewer quantities of the manufacturer’s products. Even if the manufacturer attempts to maintain profit by increasing the wholesale price and increasing revenue from the reselling channel, the platform’s order quantity continues to rise due to consumer preference. Similarly, if the third-party retailer sells a low-quality product, the utility of purchasing from the third-party retailer decreases. In contrast to the results in Scenario MD, the manufacturer reduces the wholesale price to maintain the firm’s order quantity in Scenario RD. The platform can therefore choose a larger quantity due to the higher profit margin in the reselling channel.

#### Proposition 3

When the manufacturer sells the product via the agency channel, the platform will be better off due to the product quality distribution strategy if $0<\gamma <\frac{1}{4},0<\delta <\delta_{2},0<k<k_{7}$. However, if this is comprehensively compared with the profit in Scenario B, the platform will never use the product quality distribution strategy when the manufacturer is considered to be introduced.

When the manufacturer sells through the agency channel and the commission rate is sufficiently low ($0<\gamma <\frac{1}{4}$), the product differentiation level *δ* and product cost are relatively low, and the platform prefers implementing the product quality distribution strategy. In contrast, if the commission rate or differentiation level is high, even if the manufacturer’s low-quality product increases the platform’s market share, his profit margin decreases because the wholesale price is much higher. Meanwhile, the low-quality product negatively affects the manufacturer’s profit, which also makes the platform’s profit in the agency channel decrease. Furthermore, conventional wisdom may hold that the product quality distribution strategy would then harm the manufacturer’s competitiveness, implying that the platform may earn more profit under certain conditions. However, **Proposition 3** demonstrates that when the manufacturer sells through dual channels, the product quality distribution strategy is never the best option for the platform. As previously discussed, although the low differentiation level and commission rate may benefit the platform, when compared to the results in Scenario B, it is clear that the platform will opt to sell solely through the reselling channel. This is because the platform’s additional profit from the agency channel in Scenario MD cannot offset the loss of profit caused by the platform’s shrinkage in market share compared to Scenario B.

As discussed above, although the low differentiation level and commission rate could probably benefit the platform, compared with the results in Scenario B, it is clearly found that the platform will choose the option to sell solely through the reselling channel. This is because the platform’s extra profit from the agency channel in Scenario MD cannot offset the loss of profit caused by the shrinkage of his market share compared with that in Scenario B.

#### Claim 1

*In light of the above five scenarios*, *(1) when k is low*, *Scenario B is still the best for the platform if γ is low and δ is high*. *If γ increases when the product differentiation level δ is relatively high*, *the platform will choose the manufacturer*, *but the product quality distribution strategy will not be* employed *(*i.e., *Scenario MN)*. *Otherwise*, *Scenario RD is preferred by the platform*. *(2) When k is high*, *Scenario MN will never be chosen by the platform*, *and the others will be the same in the situation where k is low*.

**Fig 2:**
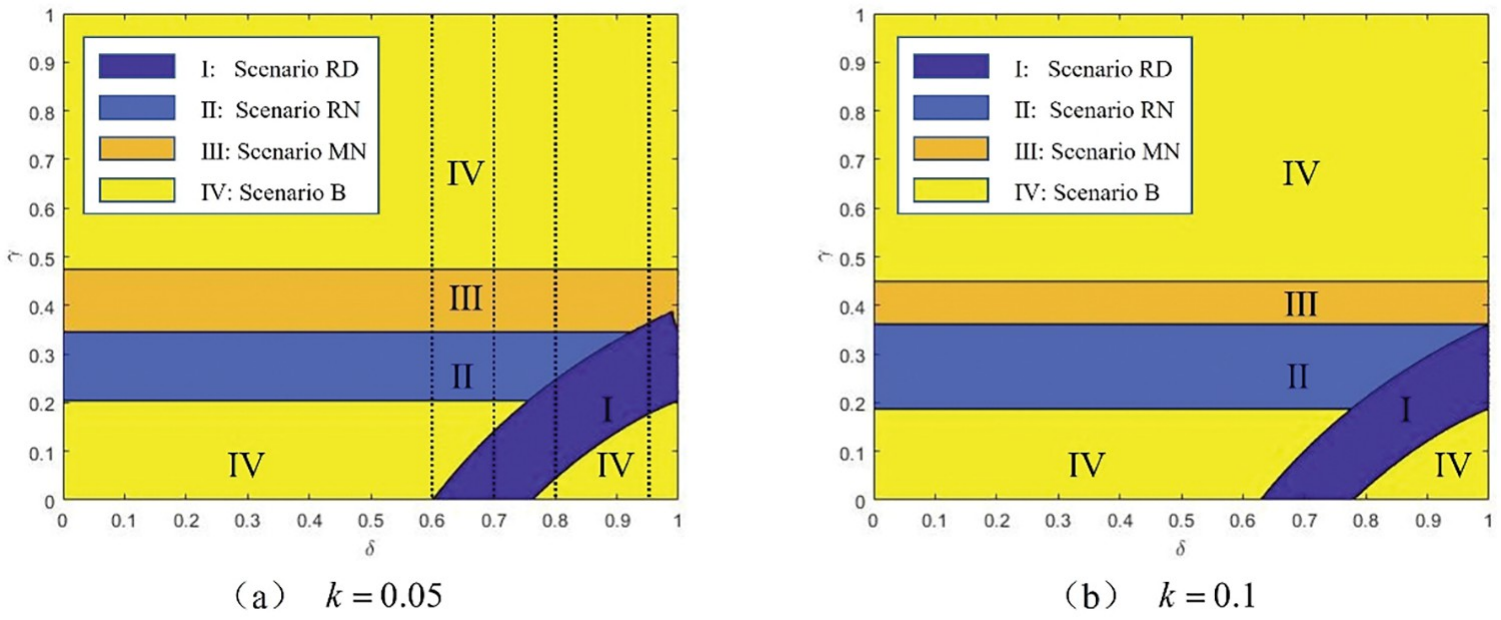
Platform’s choice of channel structure and product quality distribution strategy.

**Fig 2(A)** shows that if the production cost is sufficiently low, the selection of the manufacturer may be optimal for the platform when the product differentiation level is significantly low and the commission rate is high. In contrast, even though the agency channel and product quality distribution strategy benefit the platform to some extent, selling solely through the reselling channel is still an optimal option for the platform when the commission rate is low and the level of product differentiation is high. Additionally, in most situations, the platform prefers to choose a third-party retailer with less competition power and use the product quality distribution strategy to increase market share. Furthermore, **Fig 2(B)** shows that when the production cost is higher, the possibility of introducing a manufacturer disappears. The explanation is given below. With higher production costs, the manufacturer will set a higher wholesale price to reduce her profit as little as possible. However, in Scenario MN, the platform must bear the loss caused by the higher wholesale price alone; in Scenario RD, the third-party retailer will share the burden with him. In this case, the loss of the platform’s profit in Scenario RD is less than that in Scenario MN. As a result, rather than choosing the manufacturer, he wants to choose the third-party retailer.

**Fig 3:**
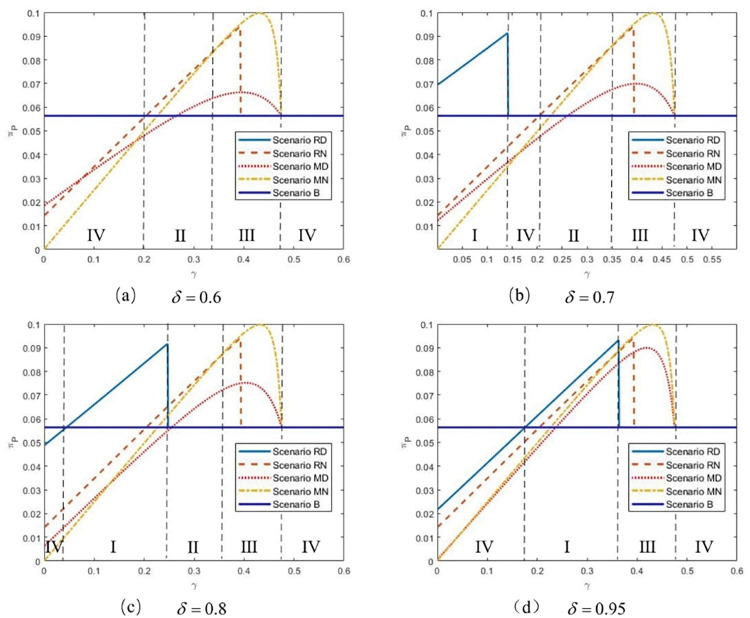
The platform’s equilibrium profits with *γ* in five scenarios, *k* = 0.05.

Following **Figs 2 and 3** chooses four typical values of *δ* (shown as dotted line in **Fig 2**) to elaborate the comparison between the platform’s profits in five scenarios with *γ* when *k* = 0.05. Intuitively, we might believe that the platform will benefit greatly from the high differentiation level (i.e., *δ* is low). However, **Fig 3** shows that the impact of the product quality distribution strategy is beyond our imagination. In particular, when the differentiation level is sufficiently high (as shown in **Fig 3(A)**), the platform cannot choose the product quality distribution strategy with any commission rate value. The basic reason is that the low-quality product severely harms the competitiveness of both the third-party retailer and the manufacturer. As a result, the third-party retailer gives up the agency channel directly. In contrast, the manufacturer can effectively deter the platform from a more aggressive wholesale price (demonstrated in **Lemma 5**). Therefore, in Scenario MD, the high differentiation level makes the platform worse off. In **Fig 3(B)**, we find that when *δ* is relatively low, the platform benefits from the product distribution when the third-party retailer sells via the agency channel with a low commission rate. However, in this situation, the third-party retailer is sensitive to the commission rate; if the commission rate exceeds a certain threshold, the retailer will withdraw from the agency channel. When the manufacturer is chosen, the relatively high value of *γ* can provide an opportunity for the product quality distribution strategy for the platform. When *δ* is intermediate and relatively high, **Fig 3(C)** and **3(D)** illustrate that the platform prefers to stay in the basic situation (Scenario B), and the agency channel and the product quality distribution strategy cannot be adopted when the commission rate is low. This is because the increase in profit from the reselling channel caused by the high-quality product is insufficient to compensate for the revenue loss from the third-party retailer. We also discover that if the differentiation level is not enough, the product quality distribution strategy is never used by the manufacturer, and the platform prefers Scenario RD and Scenario RN sequentially with a rise in *γ*. At the same time, it should be noted that the maximum value of the platform’s profit always appears in Scenario MN. The reason for this is that the manufacturer’s introduction generates enormous revenue for the platform through the agency channel and the relatively high commission rate. Meanwhile, the manufacturer has more powerful endurance than the third-party retailer. Therefore, even if the commission fee is high, she can still sell and benefit from the agency channel.

### The impact of the product quality distribution strategy on the manufacturer

Above, we examined the platform’s decision to use the product distribution approach. At the same time, the manufacturer’s preference for product distribution has drawn increasing attention, as will be explored in this section.

#### Lemma 5

Because of the product quality distribution strategy, when a third-party retailer sells the product through the agency channel, the wholesale price falls. When the product is sold by the manufacturer, the differentiated product raises the wholesale price.

According to **Lemma 5**, when a third-party retailer sells a low-quality product, the wholesale price falls. To comprehend this outcome, it is important to keep in mind that the manufacturer does not sell the product directly to customers; instead, she makes a profit by selling the original product and the low-quality product to the platform and the third-party retailer, respectively. As a result, the manufacturer’s only choice is to lower the wholesale price to make up for the decrease in the third-party retailer’s order volume brought on by the product quality distribution strategy. When the manufacturer sells the low-quality product, it is not profitable for her to sell through the agency channel. In other words, the manufacturer will set a higher wholesale price to enhance her profit margin through the reselling channel.

#### Lemma 6

*When the differentiated product is sold by the manufacturer (*i.e., *Scenario MD)*, *the manufacturer’s order quantity and the total amount are lower than* those *in Scenario MN*. In contrast, *when the third-party retailer sells via the agency channel*, *the product quality distribution strategy increases the total order quantity*.

Intuitively, **Lemma 6** indicates that the low-quality product causes the manufacturer to order fewer products in Scenario MD than in Scenario MN. Because of the lower value, the differentiated product is less appealing. As a result, the manufacturer has only to order fewer quantities of the low-quality product to minimize the total quantity of the product on the market. Based on the aforementioned decision, the price of the high-quality product rises, and the manufacturer may attempt to benefit from the reselling channel by raising the wholesale price.

#### Proposition 4

The manufacturer is worse off because of the product quality distribution strategy when she sells via the agency channel.

According to **Proposition 4**, the manufacturer’s profit drops as a result of the product quality distribution strategy. Even though the higher wholesale price allows the manufacturer to achieve high profit margins, the revenue increase from reselling is insufficient to compensate for the manufacturer’s loss due to the low-quality product. To summarize, combined with Proposition 3, we find that when the manufacturer sells through the agency channel, neither she nor the firm benefits from the product quality distribution strategy, resulting in a loss-loss conclusion.

#### Claim 2

Considering the five aforementioned situations, the manufacturer always prefers Scenario MN, in which the platform sells the product through the agency channel, while the product quality distribution strategy is not implemented.

**Fig 4:**
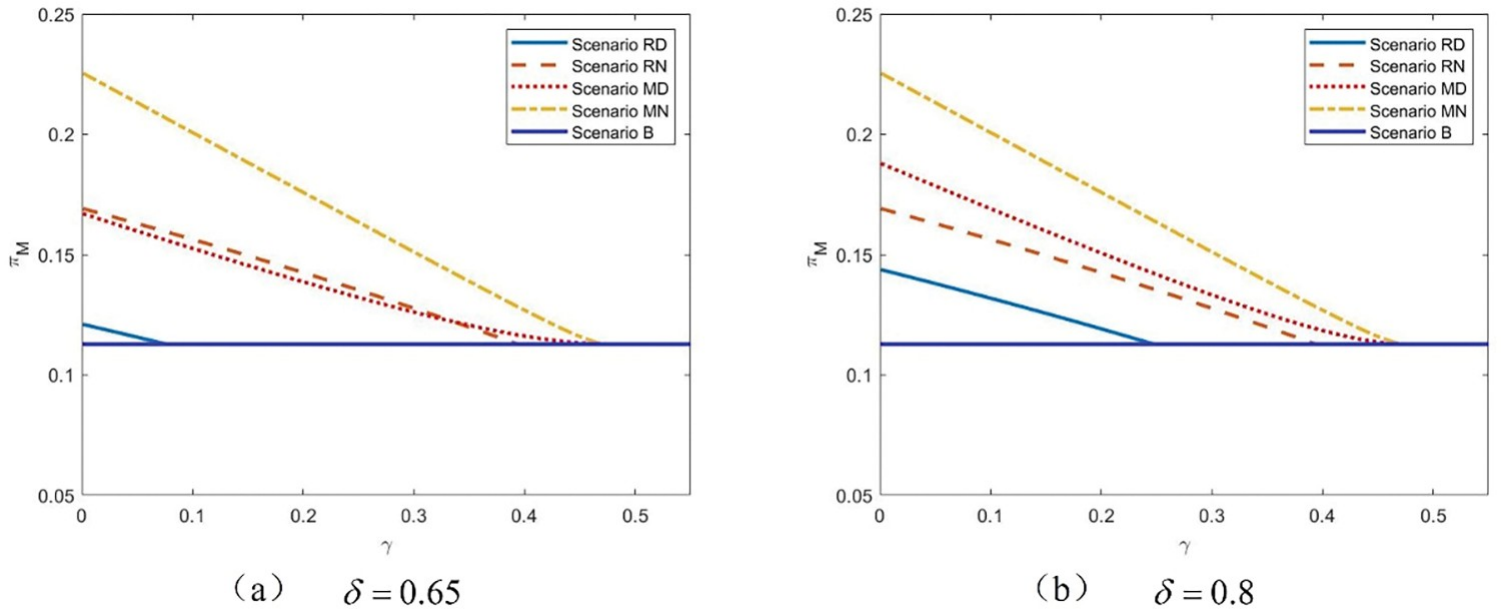
The manufacturer’s profits with *γ* in five scenarios, *k* = 0.05.

**Fig 4** illustrates the relationship between the manufacturer’s profit and the commission rate with various levels of differentiation. Surprisingly, we discover that the manufacturer always prefers Scenario MN, regardless of the differentiation level, as long as she has the option of selling through the agency channel. In Scenario MN, the manufacturer not only ignores the negative impact of the product quality distribution strategy but also has an incentive to sell more product through the agency channel. Furthermore, it is worth noting that, as opposed to the basic scenario in which the platform only sells through reselling, the hybrid retailing channel can benefit the manufacturer. This is because the introduction of a third-party company increases market competition and drives down the price of the product. Furthermore, a higher utility of purchasing the product can encourage market growth. Thus, the manufacturer’s profit increases; nevertheless, the market may be negatively impacted by the product quality distribution strategy. Furthermore, when the differentiation level rises, it appears that the manufacturer’s low-quality product weakens her competitiveness and causes her willingness to shift from Scenario MD to Scenario RN if the commission rate is low.

**Fig 5:**
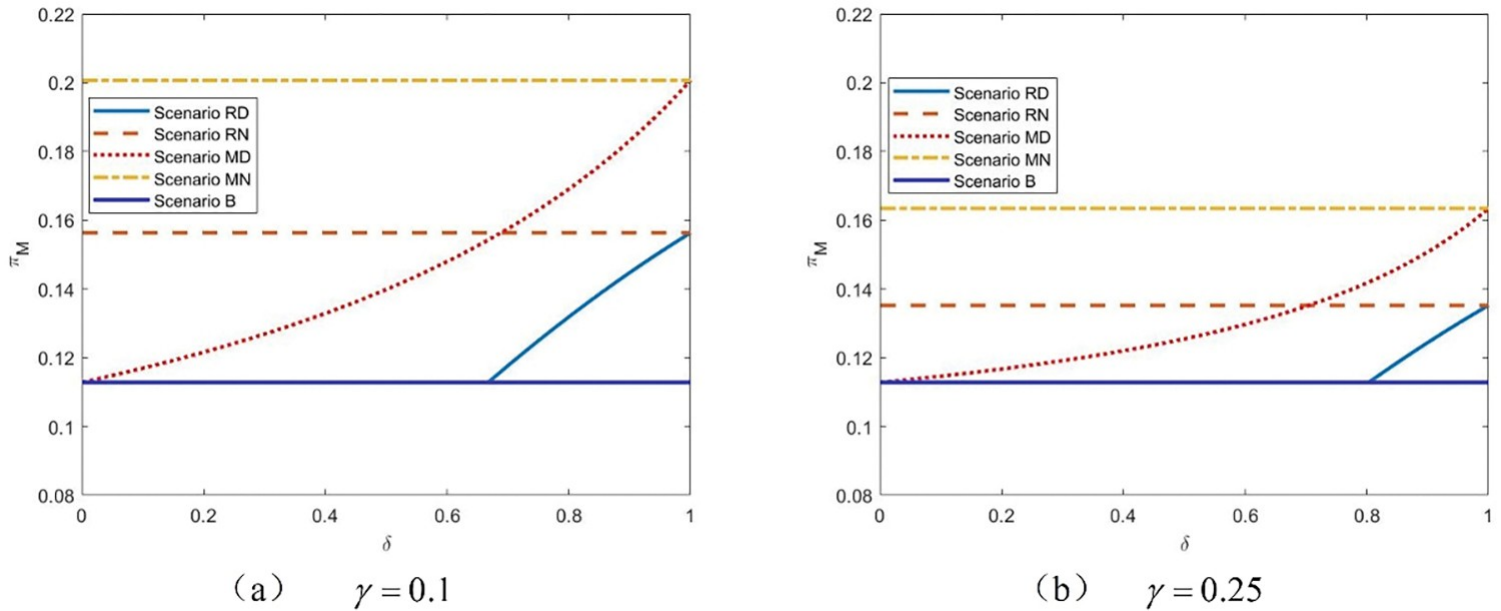
The manufacturer’s profits with *δ* in five scenarios, *k* = 0.05.

The relationship between the manufacturer’s profit and the differentiation level is further explained in **Fig 5**, which complements **Fig 4**. Because product distribution is not taken into account in Scenarios MN and RN, the manufacturer’s profits are depicted as lines in the figure. Despite the fact that **Fig 5(A)** and **5(B)** represent outcomes with various values of *γ*, the characteristics of the obvious shifting trend of the manufacturer’s profits are nearly the same. Scenario MN is always selected when the value of the commission rate is fixed. The sale of poor-quality products harms the manufacturer all the time. With a lower differential level, the manufacturer’s profit improves and approaches the value without the product quality distribution strategy. **Fig 5** further shows that, regardless of the product differentiation level, the manufacturer will accept the sale of low-quality goods in the hopes that she will subsequently choose the wholesale price to maximize her profit. However, when the third-party retailer is confronted with a product quality distribution strategy and the differentiation level is sufficient, quitting is the best alternative.

**Fig 6:**
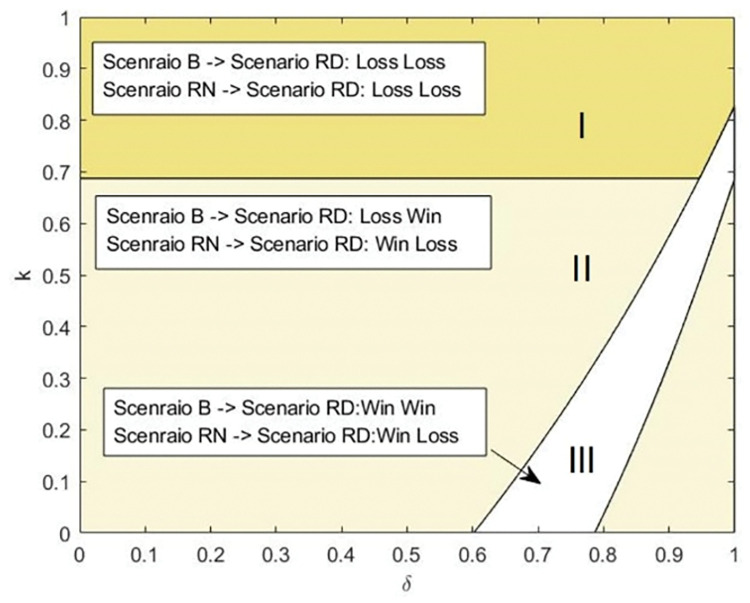
Impact of the product quality distribution strategy on the platform and the manufacturer when the third-party retailer is chosen.

If the third-party retailer is selected, **Fig 6** shows how the differential level and production cost affect the stakeholders’ profitability. As shown in Fig 6, adopting the product quality distribution strategy is unprofitable for the platform and the manufacturer when the production cost is high and the product differentiation level is low, i.e., Region I, because the third-party retailer always decides to refuse the invitation. When *k* is low, a product differentiation level that is too high or too low only favors either the platform or the manufacturer, regardless of the execution sequence of the two strategies. In Region III, if the hybrid retailing strategy and the product quality distribution strategy are implemented simultaneously, the platform and the manufacturer can achieve profit maximization given the limits of *k* and *δ*. However, if the third-party retailer is selected first, the manufacturer’s profit will suffer as the product quality distribution strategy is implemented.

**Fig 7:**
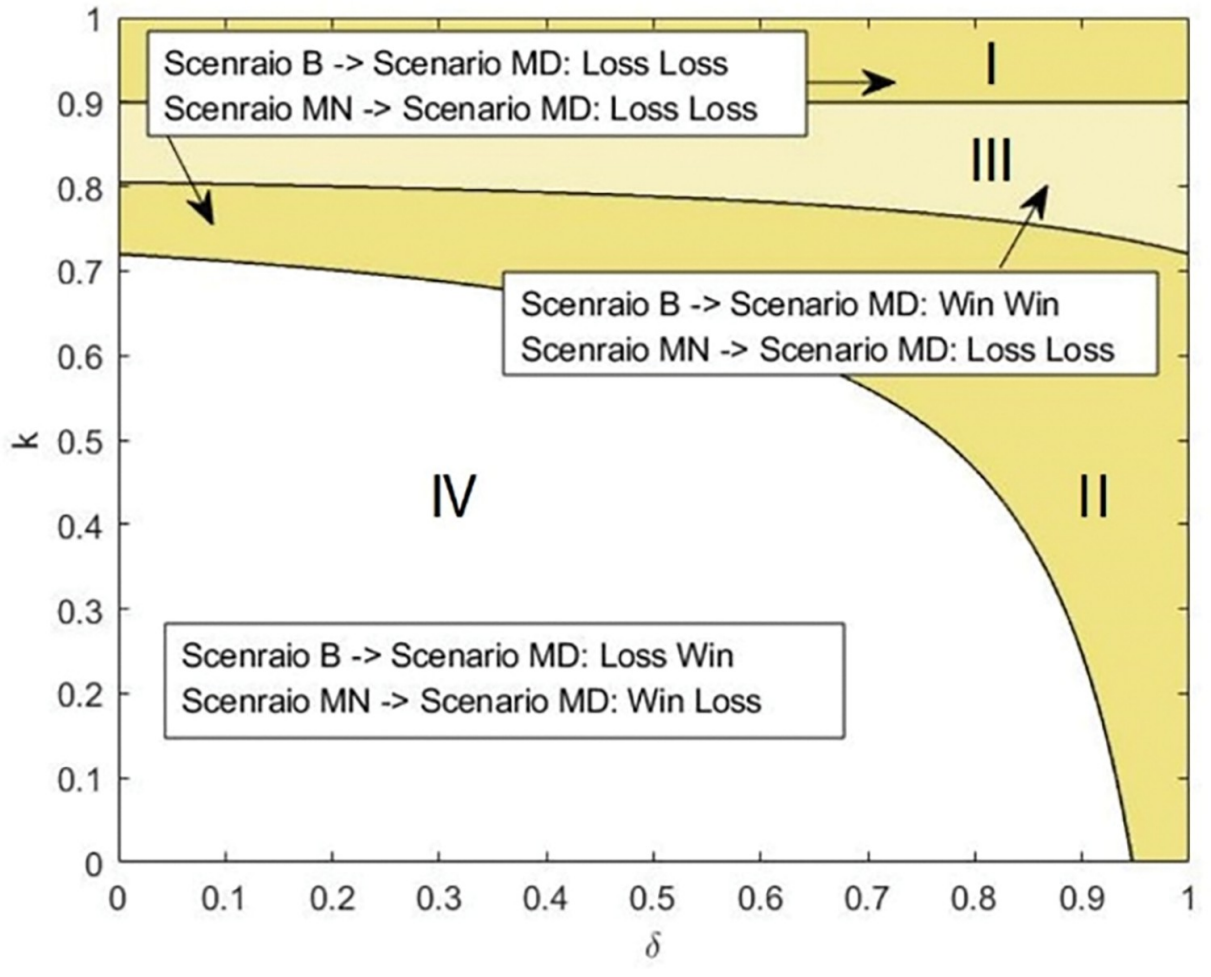
Impact of the product quality distribution strategy on the platform and the manufacturer when the manufacturer is chosen.

As shown in **Fig 7**, when the platform attempts to choose to include the manufacturer into the hybrid retailing mode, the high production cost pushes the manufacturer to quit the agency retailing mode due to the existence of a commission rate. Therefore, implementing the strategies cannot benefit the platform or the manufacturer (as shown in Region I). Even though the manufacturer in Area II is encouraged to accept the invitation by the appropriate value of the production cost, her profit is still reduced as a result of the product quality distribution strategy. In Region III, where the production cost is intermediate, if channel expansion and product distribution are adopted simultaneously, a win‒win situation is presented. Otherwise, the sequential decision results in a lose‒lose outcome. When the product differentiation level or the production cost is low (i.e., Area IV), even if the low-quality product is sold by the manufacturer, the introduction of the manufacturer reduces the platform’s profit due to the increased competition.

Overall, we discover that the sequence in which the strategies are implemented greatly affects the stakeholders’ incentives. The combined implementation is the only way to persuade the platform and the manufacturer to accept the hybrid retailing mode and product distribution. Moreover, production cost and product differentiation level also influence stakeholders’ preferences for the strategies.

## Numerical example

In order to more clearly elaborate the results, a data simulation analysis is proposed, which is similar to Zhu (2020) [38]. We assume a manufacturer who sells computer components through hybrid channel to the final customer, meanwhile the platform decides the hybrid channel structure and proposes the product quality distribution strategy. The parameters are shown as follows *s* = 2000, *δ* = {0.6, 0.8}, *γ* = [0.05, 0.15], *k* = 0.1.

Based on Tables 2 and 3, we conclude that, in Scenario RN and Scenario MN, with the rise of commission rate, the platform’s order quantity increases. Because of the higher commission rate, the third-party retailer and the manufacture orders less quantity of product respectively in Scenario RN and Scenario MN. Meanwhile the more intensive competition also forces the manufacturer to reduce the wholesale price. Therefore, the platform’s profit is better off due to the high commission rate. In contrast, the manufacturer’s and the third-party retailer’s profits is worse off. However, compared with the results in Scenario RN, we find that the platform’s order quantity and profit are less than the ones in Scenario MN, because the manufacture has the advantage of deciding wholesale price which can limit the platform’s priority.

**Table 2:** The equilibrium results in Scenario RN.

*γ*	$q_{P}^{RN}$	$q_{R}^{RN}$	*w* ^*RN*^	$\pi_{M}^{RN}$	$\pi_{P}^{RN}$	$\pi_{R}^{RN}$
0.05	0.1200	0.2162	1066	291.17	45.73	42.20
0.07	0.1234	0.2122	1052	286.02	53.87	38.95
0.09	0.1272	0.2078	1038	280.80	61.99	35.77
0.11	0.1313	0.2031	1024	275.51	70.09	32.67
0.13	0.1358	0.1980	1009	270.15	78.16	29.66
0.15	0.1407	0.1924	995	264.72	86.20	26.75

**Table 3:** The equilibrium results in Scenario MN.

*γ*	$q_{P}^{MN}$	$q_{M}^{MN}$	*w* ^*MN*^	$\pi_{M}^{MN}$	$\pi_{P}^{MN}$
0.05	0.0028	0.4444	1056	380.28	24.71
0.07	0.0041	0.4419	1038	370.41	34.56
0.09	0.0055	0.4390	1021	360.55	44.39
0.11	0.0071	0.4359	1004	350.71	54.20
0.13	0.0088	0.4324	988	340.88	63.97
0.15	0.0107	0.4286	971	331.07	73.70

Based on Table 4, we conclude that, in Scenario RD, if the product differentiation level is high (i.e., *δ* = 0.6), the third-party retailer rejects to sell product through agency channel. Thus, if the commission rate is not sufficiently low, the platform orders the quantity of product which is the same as the one in Scenario B. When the product differentiation level is low (i.e., *δ* = 0.8), the third-party retailer chooses to stay in the hybrid channel structure, which reduces the platform’s order quantity. However, with the increasing of commission rate, the third-party retailer’s market share and profit decreases, the manufacturer is also worse off.

**Table 4:** The equilibrium results in Scenario RD.

*δ*	*γ*	$q_{P}^{RD}$	$q_{R}^{RD}$	*w* ^*RD*^	$\pi_{M}^{RD}$	$\pi_{P}^{RD}$	$\pi_{R}^{RD}$
0.6	0.05	0.2250	0.0000	1100	202.50	101.25	0.00
	0.07	0.2250	0.0000	1100	202.50	101.25	0.00
	0.09	0.2250	0.0000	1100	202.50	101.25	0.00
	0.11	0.2250	0.0000	1100	202.50	101.25	0.00
	0.13	0.2250	0.0000	1100	202.50	101.25	0.00
	0.15	0.2250	0.0000	1100	202.50	101.25	0.00
0.8	0.05	0.2258	0.1108	911	243.65	109.85	10.83
	0.07	0.2303	0.1060	897	238.76	116.67	9.56
	0.09	0.2350	0.1009	884	233.81	123.50	8.36
	0.11	0.2399	0.0956	871	228.80	130.34	7.24
	0.13	0.2451	0.0900	857	223.72	137.19	6.18
	0.15	0.2505	0.0842	843	218.58	144.04	5.20

Based on Table 5, we conclude that, in Scenario MD, no matter how much the product differentiation level is, the platform still chooses to sell product through the agency channel, which is different with the third-party retailer’s decision. It is worth to notice that the lower value of *δ* and higher value of *γ* benefit the platform. Although the manufacturer is negatively affected by the higher commission rate, her earns more profit than the third-party retailer in Scenario RN with the same circumstance.

**Table 5:** The equilibrium results in Scenario MD.

*δ*	*γ*	$q_{P}^{MD}$	$q_{M}^{MD}$	*w* ^*MD*^	$\pi_{M}^{MD}$	$\pi_{P}^{MD}$
0.6	0.05	0.1327	0.3077	1082	276.35	48.00
	0.07	0.1345	0.3016	1075	271.26	53.89
	0.09	0.1365	0.2951	1068	266.24	59.72
	0.11	0.1386	0.2881	1062	261.28	65.47
	0.13	0.1408	0.2807	1056	256.39	71.14
	0.15	0.1432	0.2727	1051	251.59	76.70
0.8	0.05	0.0795	0.3636	1071	318.86	31.17
	0.07	0.0816	0.3585	1060	311.48	39.08
	0.09	0.0838	0.3529	1049	304.15	46.94
	0.11	0.0862	0.3469	1039	296.87	54.75
	0.13	0.0888	0.3404	1029	289.65	62.48
	0.15	0.0917	0.3333	1020	282.50	70.14

**Fig 8:**
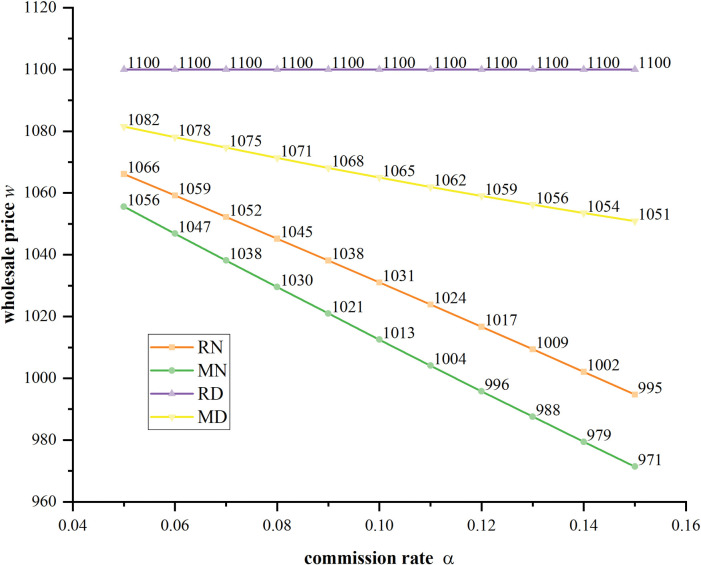
The equilibrium wholesale prices with different commission rates under four scenarios when *δ* = 0.6.

**Fig 9:**
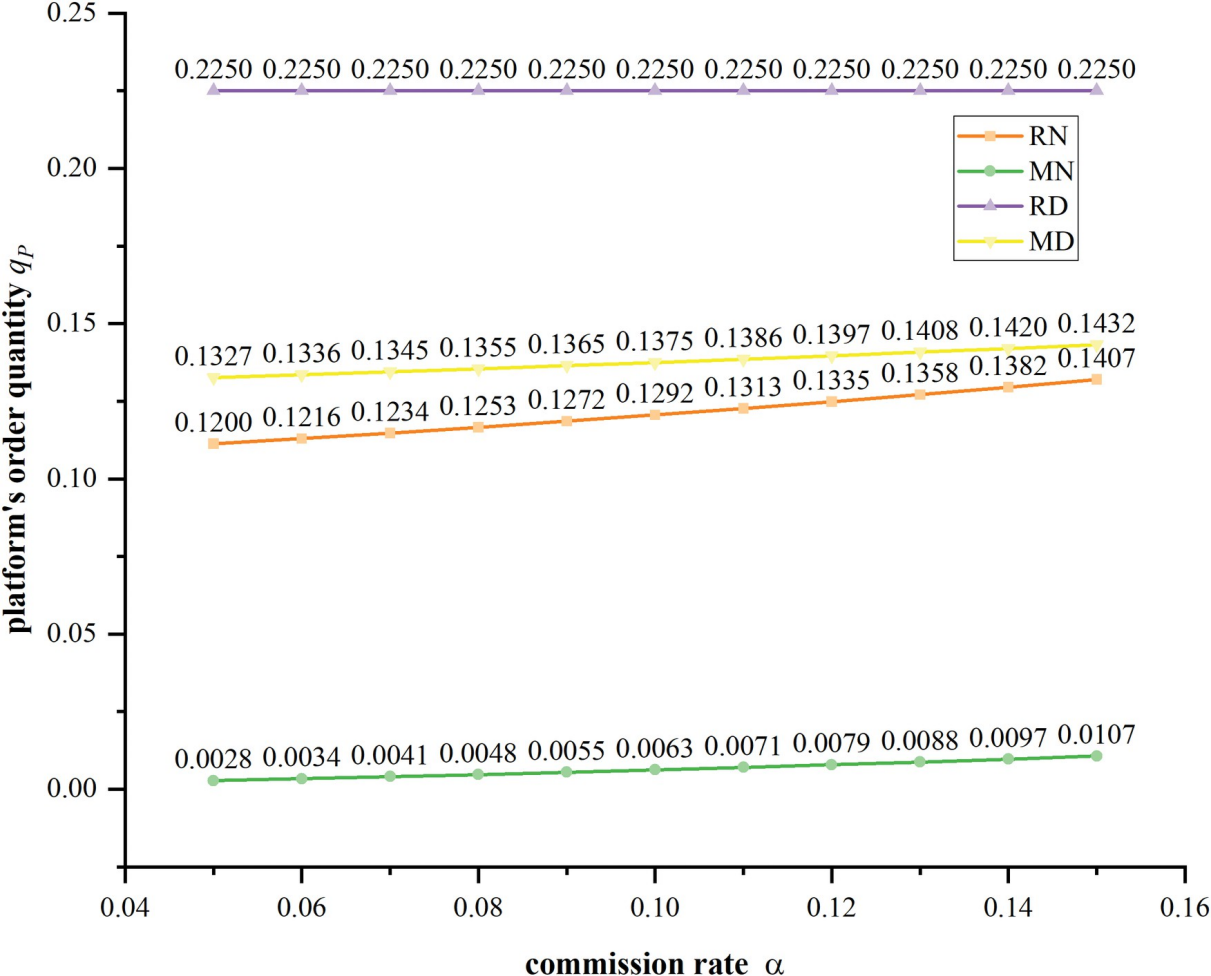
The platform’s equilibrium order quantities with different commission rates under four scenarios when *δ* = 0.6.

**Fig 8** shows that the wholesale price decreases with the increasing of commission rate in four scenarios. The manufacture sets the highest value of wholesale price in Scenario RD, because the platform solely sells in the market which reduce the competitive pressure. It is interesting to find that when the manufacture sells through the agency channel, even her sells low-quality product in Scenario MD, a higher wholesale price is decided than in Scenario MN. This is because that the manufacture wishes to increase the platform’s cost and suppress his competitiveness. **Fig 9** shows that the platform’s order quantity increases with higher commission rate. At the same time, higher market competition intensity forces the platform to sell less product. For example, in Scenario RN, the high product differentiation level makes the platform monopolize the market, so he can order the most product. In contrast, in Scenario MN, there is no product quality distribution strategy supporting the platform, and the manufacture has more competitive power, so the platform has to order the least number of products.

## Extension

### Different wholesale prices for the various retailing channels

In the main model, the manufacturer decides on a single wholesale pricing for the platform and the third-party retailer. We make this assumption because wholesale prices are often the same for all retailers. However, one may anticipate that the manufacturer will choose different wholesale prices for the platform and the third-party retailer in light of the platform’s distinct and significant role. On the one hand, it may benefit the manufacturer’s profit; on the other hand, it may benefit the platform’s and the manufacturer’s cooperation in practice. Especially, if the product quality distribution strategy is adopted, the products with different qualities also need different wholesale price. Therefore, in this subsection, we will examine whether the different wholesale prices would influence the outcomes. For the simplicity, we analyze this research problem by extending the model in Scenario RN to allow the manufacturer simultaneously and respectively decide two wholesale prices for the platform and the third-party retailer. The platform and the third-party retailer make decisions in sequence. In this section, the new scenario is named as “Scenario RNW”. The results are summarized in the following Lemma and proposition.

#### Lemma 7

When the third-party retailer sells through the agency channel, if her wholesale pricing differs from the platform’s (i.e., Scenario RNW), we find that the third-party retailer’s wholesale price decreases while the platform’s increases.

**Lemma 7** demonstrates that when the manufacturer has the option to set different wholesale prices, she will charge a higher wholesale price in order to gain from the platform, because the platform has a larger order than the third-party retailer. Meanwhile, knowing that the platform will use the option of placing a smaller order to reduce his profit loss, the manufacturer will simultaneously charge a lower wholesale price to the third-party retailer to achieve two goals: (1) maintain the order quantity, and (2) induce the third-party retailer to sell through the agency channel. The manufacturer can achieve a sustainable profit by achieving the two aims listed above.

#### Proposition 5

In comparison to the outcomes in Scenario RN, when the third-party retailer sells through the agency channel, the platform’s profit will fall but the manufacturer will benefit because of the different wholesale prices for the third-party retailer and the platform (i.e., Scenario RNW).

In contrast to the results in Scenario RN, Proposition 5 shows the effect of the different wholesale price strategy on the profitability of the stakeholders. It makes sense that the platform’s profit will decline. Because of the higher wholesale price, the platform needs order fewer units in order to raise the pricing. Although this choice may lessen the negative impact of the wholesale price increase, it cannot reverse the profit decline. The proportion of the manufacturer’s profit is altered by the different wholesale price strategy. The increased wholesale price will reduce the platform’s profitability. Simultaneously, the manufacturer can recover the loss by lowering the wholesale price for the third-party retailer and supporting the third-party retailer in increasing market share. As a result of the different wholesale price strategy, the manufacturer can gain a competitive advantage and increase profits. However, due to the scale effect, the manufacturer is usually ready to establish a lower wholesale price for the platform. Furthermore, when negotiating with the platform, the platform is more competitive and prioritizes commercial principles above the manufacturer.

### Low-quality product sold by the platform

In Scenario MD, the manufacturer is supposed to sell the low-quality product. This assumption is reasonable because the platform has priority in business with the other sellers. Therefore, when he proposes the product quality distribution strategy, it is usually profitable for the platform to sell a high-quality product. However, if the manufacturer dominates the discussion and designs the product quality distribution strategy, it may be preferable for the platform to sell a low-quality product. Thus, in this section, we will investigate whether and how a different product quality distribution strategy (i.e., the platform selling a low-quality product) affects the outcomes. We address this research issue by expanding the Scenario MN model. "Scenario MP" is the name of the new scenario. We compared the profits of stakeholders in Scenario MP to those in Scenario MN to assess the impact of the manufacturer’s proposed product quality distribution strategy. The findings are summarized in the following proposition.

#### Proposition 6

*When the product is sold by the manufacturer via the agency channel*, *compared with the results in Scenario MN*, *if the platform orders the low-quality product (*i.e., *Scenario MP)*, *the platform will be better off*. *Meanwhile*, *the manufacturer’s profit will decrease*.

**Proposition 6** illustrates that when the manufacturer sells a high-quality product, the effect on the manufacturer’s profit is the same as that in Scenario MD, in which she sells the low-quality product (as shown in **Proposition 4**), which exceeds our expectations. To mitigate the negative effect of selling a low-quantity product, the platform must limit the order quantity. Meanwhile, the manufacturer sets a lower wholesale price than in Scenario MN to maintain the platform’s order quantity. On the other hand, by selling more high-quality products, the manufacturer increases revenue from the agency channel. However, the results show that the increase in profit from the agency channel is insufficient to compensate for the loss in the reselling channel. Therefore, the manufacturer’s profit still decreases. However, when compared to the results in Scenario MD, it should be noted that a high-quality product can boost the manufacturer’s competitiveness to some extent. In contrast, the platform is better off as a result of increased revenue from the agency channel, which is also counterintuitive.

## Conclusion and managerial insights

Motivated by the observed commercial practices of platforms, we discover that platforms (for example, JD.com and Amazon.com) provide an agency channel to encourage third-party retailers and manufacturers to sell products. At the same time, as a result of the increased competition brought on by hybrid retailing, the platform will propose a specific product distribution agreement with the manufacturer. According to the agreement, the manufacturer distributes various quality products dependent on retailing channels. Most of the time, the platform sells high-quality products only through the reselling channel, while low-quality products are sold through the agency channel. The transition from traditional retailing to hybrid retailing is an opportunity for platforms. However, this is difficult for stakeholders in a highly competitive market, especially when the product quality distribution strategy is proposed at the same time. Thus, the complex influence of the hybrid retailing mode and the product quality distribution strategy is essential and meaningful to explore based on the concerns involved in these realistic scenarios.

In this paper, we have provided a deep understanding of the effects of the product quality distribution strategy under various retailing channel structures on stakeholders’ decisions. We also identify the conditions under which hybrid retailing and product distribution effectively benefit the platform and the manufacturer. To the best of our knowledge, our work is the first to examine the joint influence of the product quality distribution strategy and hybrid retailing channel choice, which extends the literature on a single hybrid retailing mode (Wei Y et al. 2022 [1]and Dai B et al. 2022 [41]). The analysis of the product quality distribution strategy under different hybrid retailing channels can provide more useful operational guidelines for the platform and the manufacturer.

In particular, we propose Stackelberg game models to investigate ordering decision problems in the context of the product quality distribution strategy with multiple channel structures. Previous research has primarily concentrated on the pricing challenge in a simple dual channel setting. In contrast, our study sheds light on how product distribution and hybrid retailing can be implemented collaboratively to benefit channel member profits, as well as how shareholders react to the product quality distribution strategy in different operational settings (e.g., product differentiation level commission rate and product cost). Thus, we examine the following five scenarios: Scenario B: the platform solely sells through the reselling channel, Scenario RN: the third-party retailer sells through the agency channel, Scenario MN: the manufacturer sells through the agency channel, Scenario RD: the third-party retailer sells the differentiated product, and the platform sells the high-quality product through the reselling channel, and Scenario MD: the manufacturer sells the low-quality product through the agency channel, and the platform exclusively sells the high-quality one.

On the basis of the equilibrium outcomes that were obtained, a comparative statics analysis is conducted to investigate the effectiveness of the product quality distribution strategy in relation to a number of different hybrid channel structures. The key findings are given here. First, when the third-party retailer sells in the hybrid channel structure, the product quality distribution strategy is only acceptable for low product differentiation levels because the double pressure from weak bargaining power and low-quality products may push the third-party retailer out of the market. When the manufacturer is chosen, however, the implementation of the product quality distribution strategy will not cause the manufacturer to abandon the agency channel. Second, under the two types of hybrid channel structures, the product quality distribution strategy can increase the platform’s order quantity. Third, even though the platform sells high-quality products, it can benefit only when a third-party retailer is introduced with a proper commission rate and product differentiation level due to the fiercer market rivalry caused by the product quality distribution strategy. Fourth, hybrid channel structures can always increase a manufacturer’s profit. However, once the agency channel is open, the manufacturer will not accept product distribution. We also add two extensions. First, we demonstrate that when the wholesale prices for the platform and the third-party retailer differ, the manufacturer erodes the platform’s profit by setting a higher wholesale price compared to the same wholesale price for market sellers. Second, if the platform sells a low-quality product, he is still better off, which is counterintuitive; in contrast, the manufacturer is worse off.

The managerial insights from this study can assist stakeholders in making decisions on product distribution and hybrid channel structures. Interestingly, for platforms, the timing of implementing a product quality distribution strategy is critical. The manufacturer will refuse the product quality distribution strategy if the agency channel is opened first. The only option is that the two strategies are implemented concurrently. Furthermore, platforms should focus more on product cost, commission rate, and product differentiation level, all of which have a significant impact on the success of the product quality distribution strategy. Even if the platform is forced to sell low-quality products, the hybrid channel structure can benefit the manufacturer in terms of profit and order quantity. We also discovered that a different wholesale pricing strategy and a high-quality product can increase the manufacturer’s profitability, which is based on the manufacturer’s strong market competitiveness. Moreover, the pricing and ordering outcomes identified in our research can help operations managers make better decisions about hybrid retailing and product distribution strategies.

The results of this study can help platforms make relative decisions. For example, when JD.com plans to introduce manufacturers (e.g., GREE, Lenovo, ASUS) to sell new product lines on the platform, the best choice is to try to sell the high-quality product while simultaneously negotiating with the manufacturers; otherwise, the manufacturers will refuse the product quality distribution strategy. Furthermore, when the product cost is high (e.g., laptops, printers, air conditioners), the manufacturer stays to sell the product through the agency channel, even if the product she sells is low-quality. Therefore, platforms can make great efforts to propose a product quality distribution strategy. Third, when JD.com decides on the commission rate of a new product, if the manufacturer is chosen, the high commission rate may not be the best choice. When the product quality distribution strategy is executed, it is appropriate to adopt a low commission rate (normally lower than 10%), which is similar to the commission rate for laptops on JD.com (i.e., 5%), where the product quality distribution strategy is executed for some kinds of laptops. Fourth, from the perspective of manufacturers (e.g., Dell, L’Oreal, Siemens), they should do all in their strength to persuade the platforms to provide hybrid retailing channels under any situation, even if only the third-party retailers sell through the agency channel. This is because the manufacturers can obtain more profits and order quantities through the agency channel.

In this paper, we concentrate on the ordering issue and assume that only one third-party retailer participates in the hybrid retailing mode. In terms of future research directions, scholars could investigate the impact of stakeholders’ market power and brand reputation on product quality distribution strategy. Another possible direction is to investigate how to use incentive contracts to gain the manufacturer’s support for the product quality distribution strategy. Furthermore, in reality, products may differ from one another in terms of quality, cost, and consumer preference; it is difficult to determine the distribution channel for each type of product, thus using big data analysis technology may be a valuable contribution.

## Supporting information

S1 File (DOCX)
