# Supplementary material for: Hybrid channel structure and product quality distribution strategy for online retail platform
Source: PLoS One. 2023 May 18;18(5):e0285860. doi: 10.1371/journal.pone.0285860 (PMC10194878; doi:10.1371/journal.pone.0285860)
Supplement: S1 File — (DOCX) [file pone.0285860.s001.docx]

# Appendix A. Analysis for Scenario RN

## A.1 Given $q_{R}$, the platform decides $q_{P}$.

Given $q_{R}$, the platform decides $q_{P}$ to maximize his profit

$\pi_{P}=(p_{p}-w)q_{p}+\gamma p_{R}q_{R}$.

From FOC, we have

## A.2 Given $w$, the third-party retailer decides $q_{R}$.

Given $w$, the third-party retailer decides $q_{R}$ to maximize her profit$\pi_{R}(q_{R})=(1-\gamma)p_{R}q_{R}-wq_{R}=(1-\gamma)s(1-q_{P}(q_{R})-q_{R})q_{R}-wq_{R}$. If , substituting into $\pi_{R}(q_{R})$ and from the FOC, we have (1) if $0\leq\gamma<3-2\sqrt{2}$, . (2) if $3-2\sqrt{2}<\gamma<1$, . If , substituting $q_{P}=0$ into $\pi_{R}(q_{R})$, from the FOC, we have (1) if , , (2) if , .

Comparing the third-party retailer’s profit, we can derive the best response as follows,

(1) if ,

(2) ,

(3) $0<\gamma<3-2\sqrt{2}$,

Substituting $q_{R}(w)$ into $q_{P}(q_{R})$, we can have $q_{P}(w)$.

## A.3 The manufacturer decides $w$.

Anticipating $q_{R}(w)$ and $q_{P}(w)$, the manufacturer can decide $w$ to maximize her profit. Based on the third-party retailer’s best response, we can have the following cases.

Case 1: For $\frac{1}{3}<\gamma<1$. (a) if , substituting $q_{R}(w)$ and $q_{P}(w)$ into $\pi_{M}$, we have . (b) if , substituting $q_{R}(w)$ and $q_{P}(w)$ into $\pi_{M}$, we have . (c) if , we find that $\pi_{M}$ is also increasing in $w$, so . (d) if , $\pi_{M}$ is still increasing in $w$, so . Comparing the four sub-cases, we have , where .

Case 2: For . (a) if $w\geq s$, substituting $q_{R}(w)$ and $q_{P}(w)$ into $\pi_{M}$, we find that $\pi_{M}$ is decreasing in $w$, so $w=s$. (b) if , substituting $q_{R}(w)$ and $q_{P}(w)$ into $\pi_{M}$, we have . (c) if , substituting $q_{R}(w)$ and $q_{P}(w)$ into $\pi_{M}$, we have . (d) if , we find that $\pi_{M}$ is increasing in $w$, so . Comparing the three sub-cases, if, we have .

Case 3. For $0<\gamma<3-2\sqrt{2}$. (a) if $w\geq s$, substituting $q_{R}(w)$ and $q_{P}(w)$ into $\pi_{M}$, we find that $\pi_{M}=0$, no matter what the value of $w$ is. (b) if , substituting $q_{R}(w)$ and $q_{P}(w)$ into $\pi_{M}$, we have . (c) if $w<\frac{(1-\gamma)s}{1+\gamma}$, substituting $q_{R}(w)$ and $q_{P}(w)$ into $\pi_{M}$, we have . Comparing the three sub-cases, if$0<\gamma<3-2\sqrt{2}$, we have .

Summarizing the above three cases, we obtain the equilibrium wholesale price as follows,

where .

Substituting $w^{TR}$ into $q_{R}(w)$, $q_{P}(w)$ and firms’ profit functions, we have

# Appendix B. Analysis for Scenario RD

## B.1 Given $q_{R}$, the platform decides $q_{P}$.

Given $q_{R}$, the platform decides $q_{P}$ to maximize his profit

$\pi_{P}=(p_{p}-w)q_{p}+\gamma p_{R}q_{R}$ s.t. $q_{p}\geq0$.

From FOC, we have

## B.2 Given $w$, the third-party retailer decides $q_{R}$.

Given $w$, the third-party retailer decides $q_{R}$ to maximize her profit

$\pi_{R}(q_{R})=(1-\gamma)p_{R}q_{R}-wq_{R}=(1-\gamma)s\delta(1-q_{P}(q_{R})-q_{R})q_{R}-wq_{R}$ s.t. $q_{R}\geq0$. If , substituting into $\pi_{R}(q_{R})$ and from the FOC, we have (1) if , , (2) if , . If , substituting $q_{P}=0$ into $\pi_{R}(q_{R})$ and from the FOC, we have (1) if , , (2) if , . Comparing the third-party retailer’s profit, we can derive the best response as follows,

(1) if , $0<\delta\leq1$,

(2) if , ,

(3) , or $\gamma<3-2\sqrt{2}$, $0<\delta\leq1$,

Substituting $q_{R}(w)$ into $q_{P}(q_{R})$, we can have $q_{P}(w)$.

## B.3 the manufacturer decides $w$.

Anticipating $q_{R}(w)$ and $q_{P}(w)$, the manufacturer can decide $w$ to maximize his profit. Based on the third-party retailer’s best response, we can have the following cases.

Case 1. For . (a) if $w>s$, we always have $q_{R}(w)=0$ and $q_{P}(w)=0$, so $\pi_{M}=0$. (b) if, substituting $q_{R}(w)$ and $q_{P}(w)$ into $\pi_{M}$, we have . (c) if , substituting $q_{R}(w)$ and $q_{P}(w)$ into $\pi_{M}$, we have , . (c) if , we find that $\pi_{M}$ is also increasing in $w$, so . (d) if , $\pi_{M}$ is still increasing in $w$, so . Comparing the four sub-cases, we have , where ,

Case 2: For ,. (a) if $w\geq s$, substituting $q_{R}(w)$ and $q_{P}(w)$ into $\pi_{M}$, we find that $\pi_{M}$ is decreasing in $w$, so $w=s$. (b) if , substituting $q_{R}(w)$ and $q_{P}(w)$ into $\pi_{M}$, we have. (c) if , substituting $q_{R}(w)$ and $q_{P}(w)$ into $\pi_{M}$, we have

. (d) if , we find that $\pi_{M}$ is increasing in $w$, so . Comparing the four sub-cases, if, , we have

.

Case 3. For , or $\gamma<3-2\sqrt{2}$. (a) if $w\geq s$, substituting $q_{R}(w)$ and $q_{P}(w)$ into $\pi_{M}$, we find that $\pi_{M}=0$, no matter what the value of $w$ is. (b) if , substituting $q_{R}(w)$ and $q_{P}(w)$ into $\pi_{M}$, we have

. (c) if , substituting $q_{R}(w)$ and $q_{P}(w)$ into $\pi_{M}$, we have . Comparing the three sub-cases, if, or $\gamma<3-2\sqrt{2}$, we have

According to the above three cases, we have

where $k^{RD}=k_{2}^{RD}$. Substituting $w^{TR}$ into $q_{R}(w)$, $q_{P}(w)$ and firms’ profit functions, we have

# Appendix C. Analysis for Scenario MD.

## C.1 Given $w$ and $q_{M}$, the platform decides $q_{P}$.

Given $w$ and $q_{M}$, the platform decides $q_{P}$ to maximize his profit $\pi_{P}=(p_{P}-w)q_{P}+\gamma p_{M}q_{M}$, s.t. $p_{p}\geq0$, where $p_{P}=s(1-q_{P}-\delta q_{M})$ and $p_{M}=\delta s(1-q_{P}-q_{M})$.

From FOC, we have the platform’s best response .

## C.2 The manufacturer decides $w$ and $q_{M}$.

Based on $q_{P}(w,q_{M})$, the manufacturer decides $w$ and $q_{M}$ to maximize her profit $\pi_{M}(q_{M},w)=((1-\gamma)p_{M}-k\delta s)q_{M}+(w-ks)q_{P}$, s.t. $q_{M}\geq0$. Substituting into $\pi_{M}(q_{M},w)$, from the FOC, we have

where $k^{MD}=k^{MN}=1-2\gamma$.

Substituting $w^{MD}$ and $q_{M}^{MD}$ into $q_{P}(w,q_{M})$ and firms’ profit functions, we have

# Appendix D. Proofs

**Proof of Lemma 1.** (a) For or $k^{RN}<k<1$, $q_{R}^{RN}=0$, . (b) For and $0<k<k^{RN}$, , which is decreasing in $k$. If $k_{1}<k<k^{RN}$, where , $q_{P}^{B}<q_{P}^{RN}$. Otherwise, if $0<k<k_{1}$, $q_{P}^{B}>q_{P}^{RN}$.

**Proof of Lemma 2.** For , . Thus, $q_{P}^{B}<Q^{RN}$.

**Proof of Lemma 3**. (a) For $0<\gamma<\frac{1}{2},0<k<k^{MN}$, . Thus, $q_{P}^{B}>q_{P}^{MN}$. (b) If $0<\gamma<\frac{1}{2},0<k<k^{MN}$, , if and only if ; otherwise, if $k_{2}<k<k^{MN}$, $q_{P}^{MN}>q_{M}^{MN}$.

**Proof of Lemma 4.** For $0<\gamma<\frac{1}{2},0<k<k^{MN}$, . Thus, $q_{P}^{B}<Q^{MN}$.

**Proof of Proposition 1. (a)** For $0<\gamma\leq\frac{3}{7},0<k<k^{RN}$,. The is the solution to . If $k_{3}\in(0,k^{RN})$, we have which is the solution to $\left\{\begin{matrix} k_{3}>0 \\ k_{3}<k^{RN} \end{matrix}\right.$. Thus, if or $0<\gamma<\gamma_{1},k_{3}<k<k^{RN}$ the platform switches to the hybrid channel and the third-party retailer is agree to sells the product via the agency channel, $\pi_{P}^{B}<\pi_{P}^{RN}$. Otherwise, if $0<\gamma<\gamma_{1},0<k<k_{3}$, $\pi_{P}^{B}>\pi_{P}^{RN}$, which means that the platform will not introduce the third-party retailer and solely sells through the reselling channel.

**(b)** For $0<\gamma<\frac{1}{2},0<k<k^{MN}$, .

The $k<k_{4}=8\gamma^{2}-6\gamma+1$ is the solution to . If $k_{4}\in(0,k^{MN})$, we have $\gamma<\frac{1}{4}$ which is the solution to $\left\{\begin{matrix} k_{4}>0 \\ k_{4}<k^{MN} \end{matrix}\right.$. Thus, if or , we have $\pi_{P}^{B}<\pi_{P}^{MN}$, the manufacturer will sells via the agency channel, which is accepted by the platform. Otherwise, if , we have $\pi_{P}^{B}>\pi_{P}^{MN}$, the platform will still sell through the reselling channel.

**(c)** In order to simultaneously consider the option to introduce the third-party retailer and the one to introduce the manufacturer, is considered. When , we always have , therefore $k\in(0,k^{RN})$ is the range used to analyze the choice of the platform on the firm chosen. So, for , .

The is the solution to $\pi_{P}^{RN}-\pi_{P}^{MN}<0$. If $k_{5}\in(0,k^{RN})$, we have , where $\gamma_{2}\approx0.3756$ is the solution to $k^{RN}(\gamma_{2})=k_{5}(\gamma_{2})$. makes sure that the $k_{5}$ is not an imaginary number. Therefore, if or , we have $\pi_{P}^{RN}<\pi_{P}^{MN}$. Simultaneously considering Proposition 1(a), the platform will select the hybrid channel and choose the manufacturer (i.e., $\pi_{P}^{MN}=\max(\pi_{P}^{B},\pi_{P}^{RN},\pi_{P}^{MN})$), if and only if ,. In contrast, if or , we have $\pi_{P}^{RN}>\pi_{P}^{MN}$. Based on Proposition 1(b), if $0<\gamma<\gamma_{1},k_{3}<k<k^{RN}$ or or , the choice of the third-party retailer is the best strategy for the platform (i.e., $\pi_{P}^{RN}=\max(\pi_{P}^{B},\pi_{P}^{RN},\pi_{P}^{MN})$).

**Proof of Lemma 5. (i)** For  and $0<k<k^{RN}$, we always have. Thus, the manufacturer’s wholesale price will decrease responding to the choice of the third-party retailer. **(ii)** For and $0<k<k^{MN}$, . So, even the platform chooses the manufacturer to be selected, the manufacturer will still decrease the wholesale price. (iii) In summary, because $w^{B}=\max(w^{B},w^{RN},w^{MN})$, the manufacturer will decide the largest wholesale price in Scenario B.

**Proof of Proposition 2. (a)** (i) For and $0<k<k^{RN}$, , if and only if . Because $0<k<k^{RN}$, we always have $\pi_{M}^{B}<\pi_{M}^{RN}$. So the manufacture will be better off if the platform introduces the third-party retailer. (ii) For and $0<k<k^{MN}$, , which means that the manufacturer prefers the hybrid channel format, where she can sell directly via the agency channel.

**(b)** As discussed in **Proof of Proposition 1(c)**, in order to compare Scenario RN and Scenario MN, we focus on the range and $0<k<k^{RN}$, . When , we always have $\pi_{M}^{RN}-\pi_{M}^{MN}<0$. Comprehensively consider the results in Proposition 2(a), we have $\pi_{M}^{MN}=\max(\pi_{M}^{B},\pi_{M}^{RN},\pi_{M}^{MN})$, if and $0<k<k^{RN}$. It is indicated that the manufacturer always prefers to directly sell via the agency channel than selling to the third-party retailer or only to the platform.

**Proof of Lemma 4. (i)** The third-party retailer sells the low-quality product. For*,* and $0<k<k^{RD}$, . is the solution to $q_{P}^{RD}-q_{P}^{RN}>0$. If $k_{6}>k^{RD}$, we have , where . According to , it is always true that , and . Thus, . So, $k_{6}>k^{RD}$ is demonstrated, we have $q_{P}^{RD}>q_{P}^{RN}$. **(ii)** The manufacture sells the low-quality product. For, $0<\delta\leq1$ and $0<k<k^{MD}$, . Under the conditions shown above, we always have $q_{P}^{MD}>q_{P}^{MN}$.

**Proof of Proposition 3. (i)** When the manufacturer sells the low-quality product through the agency channel, for $0<\gamma<\frac{1}{2}$, $0<\delta\leq1$ and $0<k<k^{MD}$, . The $k<k_{7}$ is the solution to $\pi_{P}^{MD}-\pi_{P}^{MN}>0$, where . If $k_{7}\in(0,k^{MD})$, we have , $0<\delta<\delta_{2}$ is the solution to $\left\{\begin{matrix} k_{7}>0 \\ k_{7}<k^{MD} \end{matrix}\right.$, where . Thus, if , we have $\pi_{P}^{MD}>\pi_{P}^{MN}$. **(ii)** Firstly, we compare the manufacturer’s profit in Scenario MD with the one in Scenario B, for , $0<\delta\leq1$ and $0<k<k^{MD}$, . The $k<k_{8}$ is the solution to $\pi_{P}^{B}-\pi_{P}^{MD}>0$, where . If $k_{8}\in(0,k^{MD})$, we have and , we have . Thus, if or , we have $\pi_{P}^{B}>\pi_{P}^{MD}$. Secondly, simultaneously consider the results discussed in the first part of this proof, we find that when $\pi_{P}^{MD}>\pi_{P}^{MN}$, the conditions should satisfy , which also is a subset of the conditions resulting in $\pi_{P}^{B}>\pi_{P}^{MD}$. In summary, we have the conclusion that if comprehensively comparing with the profit in Scenario B, the platform will never adopt the product quality distribution strategy when the manufacturer is considered to be chosen.

**Proof of Lemma 5. (i)** Considering that the third-party retailer is chosen, for *,* and $0<k<k^{RD}$, . The $k>k_{9}$ is the solution to $w^{RD}-w^{RN}>0$, where . Because *,* , it is always true that $k_{9}<0$. Therefore, when *,* and $0<k<k^{RD}$, we have $w^{RD}>w^{RN}$. **(ii)** If the manufacturer sells product, for , $0<\delta\leq1$ and $0<k<k^{MD}$, . It is obvious that $w^{MD}-w^{MN}<0$. Therefore, we have the conclusion that when the product is sold by the manufacturer, the differentiated product will improve the wholesale price.

**Proof of Lemma 6. (i)** For , $0<\delta\leq1$ and $0<k<k^{MD}$, . We can clearly find that . So, when , $0<\delta\leq1$ and $0<k<k^{MD}$, $q_{M}^{MD}<q_{M}^{MN}$. **(ii)** For *,* and $0<k<k^{RD}$, . Under the conditions, we always have $q_{P}^{RD}+q_{R}^{RD}-q_{P}^{RN}-q_{R}^{RN}>0$.

**Proof of Proposition 4.** For , $0<\delta\leq1$, $0<k<k^{MD}$,. Because $s>0$, we always have . Therefore, $\pi_{M}^{MD}<\pi_{M}^{MN}$.

**Proof of Lemma 7. (i)** Analysis on the Scenario RNW. In Scenario RNW, the manufacturer, the platform and the third-party retailer sequentially make decisions to maximize profit based on the following objective functions: $\pi_{M}(w_{R},w_{P})=w_{R}q_{R}+w_{P}q_{P}-ks(q_{R}+q_{P})$, $\pi_{R}(q_{R})=(1-\gamma)p_{R}q_{R}-w_{R}q_{R}$ s.t. $q_{R}\geq0$ and $\pi_{P}(w_{p})=(p_{p}-w_{p})q_{p}+\gamma p_{R}q_{R}$ s.t. $q_{P}\geq0$. We backward solve the game for the equilibrium. When the platform and the third-party retailer sell the products respectively via the reselling channel and the agency channel, we have the following equilibrium results: , , and . The necessary but not sufficient conditions are $0<\gamma<2-\sqrt{3},0<\delta\leq1,0<k<k^{RNW}$, where $k^{RNW}=1-2\gamma$. **(ii)** In order to compare the results in Scenario RN and Scenario RNW when the third-party retailer sells via the agency channel, is considered. When $\gamma\in(0,2-\sqrt{3})$, we have , therefore $k\in(0,k^{RN})$ is the range used. **(iii)** So, for $0<\gamma<2-\sqrt{3}$,$0<k<k^{RN}$, we always have and . Therefore, we conclude that if the manufacturer decides different wholesale prices, the third-party retailer’ wholesale price will decrease, in contrast the platform’s profit will increase.

**Proof of Proposition 5.** Based on the equilibrium results in the **Proof of Lemma 7(i)**, for $0<\gamma<2-\sqrt{3},0<\delta\leq1,0<k<k^{RNW}$,we derive the manufacturer’s and the platform’s equilibrium profits as follows, , . Thus, and

. When $0<\gamma<2-\sqrt{3},0<\delta\leq1,0<k<k^{RNW}$, we always have $\pi_{P}^{RN}-\pi_{P}^{RNW}>0$ and $\pi_{M}^{RN}-\pi_{M}^{RNW}<0$.

**Proof of Proposition 6. (i)** Analysis on the Scenario MP. In Scenario MP, the product quality distribution strategy is proposed and the low-quality product is sold by the platform. So, the manufacturer and the platform sequentially make decisions to maximize themselves’ profits. Firstly, the manufacturer decides wholesale price and order quantity. Secondly, the platform decides the order quantity. The manufacturer’s and the platform’s profit functions are shown as follows. $\pi_{M}(w,q_{M})=wq_{P}+(1-\gamma)p_{M}q_{M}-ksq_{M}-k\delta q_{P}$ s.t. $q_{M}\geq0$. $\pi_{P}(q_{P})=(p_{P}-w)q_{P}+\gamma p_{M}q_{M}$ s.t. $q_{P}\geq0$. The prices of the products sold by the platform and by the manufacturer respectively are $p_{P}=\delta s(1-q_{P}-q_{M})$ and $p_{M}=s(1-\delta q_{P}-q_{M})$. We backward solve the game for the equilibrium. When the manufacturer chooses to sell via the agency channel, we have the following equilibrium results: , , . The necessary but not sufficient conditions include (1) ; (2) , where . Based on the equilibrium decisions above, we can get the stakeholders’ profits equilibrium as follows: , . **(ii)** In order to compare the results in Scenario MN and Scenario MP when the platform sells via the agency channel, is considered. When and $\delta\in(0,1]$, we have , therefore $k\in(0,k^{MN})$ is the range used. **(iii)** So, for , we always have , . Therefore, we conclude that when the platform is chosen, if the platform orders the low-quality product, the platform is better off, the manufacturer’s profit decreases.

# Appendix E. Scenarios in Practice

Table E.1 shows the five scenarios in practice.

**Table E.1** Scenarios in practice

| **Product Category** | **Manufacturer** | **Platform** | **Product line** | **Scenario** |
| --- | --- | --- | --- | --- |
| Screen and Computer | Microsoft | JD.com | Surface Hub 2S | B^1^ |
| Phone | Apple | JD.com | iPhone | RN^2^ |
| Piano | Pearl River | JD.com | J6 | MN^3^ |
| Laptop | Microsoft | JD.com | Surface Laptop Studio | RD^4^ |
| Laptop | Lenovo | JD.com | Xiaoxin air14 | MD^5^ |

**Notes:**1. Scenario B means that Surface Hub 2S produced by Microsoft is only sold by JD.com through the reselling channel. The manufacturer and third-party retailers don’t sell this kind of product in JD.com through agency channel.

2. Scenario RN means that iPhones produced by Apple are simultaneously sold by JD.com through the reselling channel and the third-party retailers through the agency channel. The iPhones sold by the above agents have the same quality.

3. Scenario MN means that the J6 series of pianos produced by Pearl River are simultaneously sold by JD.com through the reselling channel and the manufacturer through the agency channel. The pianos sold by the above agents have the same quality.

4. Scenario RD means that Surface Laptop Studio is sold by JD.com through the reselling channel and the third-party retailer through the agency channel. However, the product sold by JD.com has a higher quality on GPU than the one sold by the third-party retailer.

5. Scenario MD means that the laptop Xiaoxin air14 is sold by JD.com through the reselling channel and the manufacturer through the agency channel. However, the product sold by JD.com has a higher quality on SSD than the one sold by the manufacturer.
